# Interferon-induced HERC5 is evolving under positive selection and inhibits HIV-1 particle production by a novel mechanism targeting Rev/RRE-dependent RNA nuclear export

**DOI:** 10.1186/1742-4690-11-27

**Published:** 2014-04-03

**Authors:** Matthew William Woods, Jessica Gayle Tong, Sean Kevin Tom, Peter Anthony Szabo, Peter Craig Cavanagh, Jimmy Dimitrios Dikeakos, SM Mansour Haeryfar, Stephen Dominic Barr

**Affiliations:** 1 Department of Microbiology and Immunology, Dental Sciences Building Room 3006b, The University of Western Ontario, Schulich School of Medicine and Dentistry, Center for Human Immunology, London, Ontario, Canada

**Keywords:** HERC5, HIV, Retroviruses, Interferon, Restriction, Antiviral, E3 ligase, Positive selection, Nuclear export

## Abstract

**Background:**

Type I interferon (IFN) inhibits virus replication by activating multiple antiviral mechanisms and pathways. It has long been recognized that type I IFNs can potently block HIV-1 replication *in vitro*; as such, HIV-1 has been used as a system to identify and characterize IFN-induced antiviral proteins responsible for this block. IFN-induced HERC5 contains an amino-terminal Regulator of Chromosome Condensation 1 (RCC1)-like domain and a carboxyl-terminal Homologous to the E6-AP Carboxyl Terminus (HECT) domain. HERC5 is the main cellular E3 ligase that conjugates the IFN-induced protein ISG15 to proteins. This E3 ligase activity was previously shown to inhibit the replication of evolutionarily diverse viruses, including HIV-1. The contribution of the RCC1-like domain to the antiviral activity of HERC5 was previously unknown.

**Results:**

In this study, we showed that HERC5 inhibits HIV-1 particle production by a second distinct mechanism that targets the nuclear export of Rev/RRE-dependent RNA. Unexpectedly, the E3 ligase activity of HERC5 was not required for this inhibition. Instead, this activity required the amino-terminal RCC1-like domain of HERC5. Inhibition correlated with a reduction in intracellular RanGTP protein levels and/or the ability of RanGTP to interact with RanBP1. Inhibition also correlated with altered subcellular localization of HIV-1 Rev. In addition, we demonstrated that positive evolutionary selection is operating on HERC5. We identified a region in the RCC1-like domain that exhibits an exceptionally high probability of having evolved under positive selection and showed that this region is required for HERC5-mediated inhibition of nuclear export.

**Conclusions:**

We have identified a second distinct mechanism by which HERC5 inhibits HIV-1 replication and demonstrate that HERC5 is evolving under strong positive selection. Together, our findings contribute to a growing body of evidence suggesting that HERC5 is a novel host restriction factor.

## Background

The cellular HERC5 protein was recently identified as an antiviral protein that inhibits replication of evolutionarily diverse viruses [1-3]. HERC5 belongs to a family of 6 HERC proteins containing an amino-terminal RCC1-like domain, a spacer region that does not share homology with any known protein, and a carboxyl-terminal HECT domain. Phylogenetic analysis of the *HERC* family revealed that the *HERC4* ancestor emerged in nematodes and that the *HERC* family expanded to six members during animal evolution, with *HERC5* being the most recently emerged family member [4]. HERC5 is ubiquitously expressed in many cell types and tissues including, but not limited to, effector and central memory T cells, dendritic cells, CD14+ monocytes, monocyte-derived macrophages, embryonic and induced pluripotent stem cells, hematopoietic and granulopoietic stem cells, testis (germ and leydig cells), ovary, liver and lung [5-17]. HERC5 expression is up-regulated in response to IFN [18,19], *in vitro* and *in vivo* virus infection [1,20-25], lipopolysaccharide, tumor necrosis factor α, and interleukin-1β [26].

HERC5 is the main cellular E3 ligase that conjugates the ubiquitin-like protein ISG15 to proteins in human cells via a hierarchical enzymatic cascade involving E1 activating enzyme Ube1L and E2 conjugating enzyme UbcH8 [18,19]. The conjugation of ISG15 to proteins is commonly referred to as ISGylation. We previously showed that HERC5 inhibits HIV-1 Gag particle assembly by a mechanism correlating with the post-translational modification of Pr55Gag with ISG15 [1]. HERC5 inhibits influenza A virus replication by catalyzing the conjugation of ISG15 to the viral NS1 protein, thereby preventing NS1 from forming homodimers and inhibiting corresponding antiviral processes [2]. Furthermore, HERC5 conjugates ISG15 to the human papillomavirus (HPV) L1 capsid protein, conferring a dominant-inhibitory effect on the infectivity of HPV16 pseudoviruses [3].

Although much is known about the HECT domain of HERC5 and its critical role in E3 ligase activity, little is known about the contribution of the RCC1-like domain region to the antiviral activity of HERC5. Proteins such as HERC5 that contain RCC1-like domains belong to a phylogenetically widespread RCC1 superfamily of proteins [27,28]. The prototypical member of this superfamily is human RCC1. RCC1 is characterized by the presence of 7 repeats of ~60 amino acids in length that assume a 7-bladed β-propeller structure. RCC1 is localized in the nuclei of eukaryotic cells where it binds and activates the GTPase Ras-related nuclear (Ran) protein [29,30]. Subsequent hydrolysis of GTP to guanosine diphosphate (GDP) by its intrinsic GTPase activity returns Ran to an inactive state. RCC1 maintains a higher level of RanGTP in the nucleus compared to the cytoplasm (>1000-fold), which is critical for Crm1-dependent nuclear export of macromolecules [31].

Here we present the molecular characterization of a second distinct and novel antiviral function of HERC5. This function targets HIV-1 Rev/RRE function and involves the RCC1-like domain of HERC5. We also demonstrate that HERC5 is evolving under strong positive evolutionary selection. Together, these observations provide new insight into the innate immune response towards HIV-1.

## Results

### HERC5 inhibits HIV-1 particle production by an E3 ligase-independent mechanism

**Figure 1:**
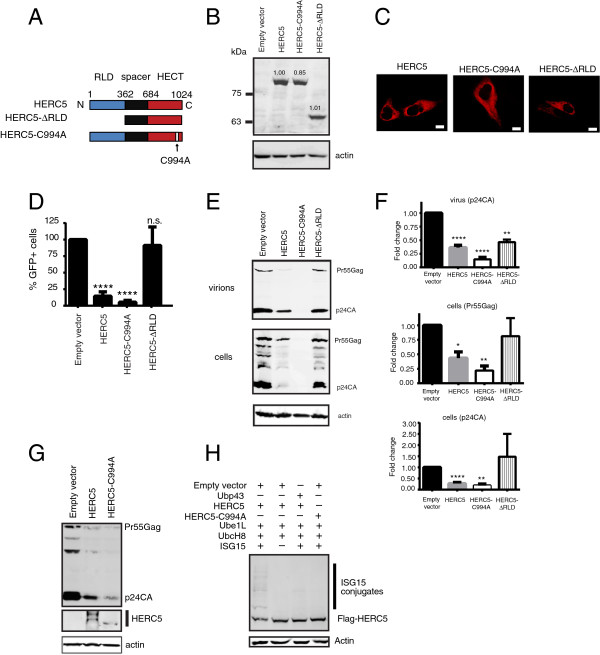
**HERC5 inhibits HIV-1 particle production via an E3 ligase-independent mechanism. A**, Schematic of HERC5 domains and the various mutant constructs generated (not to scale). **B**, Western blot analysis of 293T cells transfected with either empty plasmid or a plasmid encoding flag-tagged HERC5, HERC5-C994A or HERC5-∆RLD. Anti-flag was used to detect HERC5 and anti-β-actin was used as a loading control. Numbers above bands represent densitometric quantification relative to wildtype HERC5 after normalization to β-actin. **C**, U2OS cells were transfected as described in panel B. Forty-eight hours post-transfection, cells were analyzed by confocal immunofluorescence microscopy using anti-flag. Scale bars = 10 μm. **D**, 293T cells were co-transfected with pR9 and either empty plasmid, pHERC5, pHERC5-C994A, or pHERC5-∆RLD. Forty-eight hours after transfection, infectious virions released into the supernatant were quantified using GHOST(3) indicator cells. The averages +/- SD from at least three independent experiments are shown. **E**, Forty-eight hours after 293T cells were transfected as described in panel D, virions released into the supernatant and Gag within cell lysates were analyzed by Western blotting using anti-p24CA or anti-β-actin. **F**, Densitometric analysis of the indicated bands relative to the empty vector control from at least three independently generated experiments similar to that shown in panel E. Values were normalized to β-actin. ****P < 0.0001; **P < 0.01; *P < 0.05; n.s. P > 0.05 (student’s paired t test). **G**, Western blot analysis of 293T cells co-transfected with pR9 and either empty plasmid, flag-tagged pHERC5 or pHERC5-C994A. Forty-eight hours after transfection, cell lysates were analyzed using anti-p24CA, anti-flag or anti-β-actin. **H**, Cells were co-transfected with pUbe1L, pUbcH8 and myc-tagged ISG15 and either empty vector, pUbp43, flag-tagged pHERC5 or pHERC5-C994A. Cell lysates were analyzed by Western blotting using anti-flag or anti-β-actin. Data shown is representative of at least three independent experiments.

The E3 ligase activity of HERC5 was previously shown to contribute to the inhibition of Pr55Gag particle production [1]. To examine the contribution of the RCC1-like domain to inhibition of infectious HIV-1 particle production, we generated flag-tagged HERC5 constructs lacking the RCC1-like domain (HERC5-ΔRLD) or lacking E3 ligase activity (HERC5-C994A) (Figure 1A). Cysteine 994 is essential for HERC5-induced ISGylation [18]. Quantitative Western blot analysis of cell lysates showed that all HERC5 constructs exhibited similar levels of intracellular protein expression (Figure 1B). The HERC5 constructs were also analyzed by confocal immunofluorescence microscopy and found to be localized in the cytoplasm of U2OS cells, similar to wild type HERC5 (Figure 1C). Similar localization was observed in 293T and HeLa cells (data not shown).

We then tested the ability of these HERC5 constructs to inhibit HIV-1 replication. 293 T cells were co-transfected with plasmids encoding full-length, replication-competent HIV-1 (pR9) and either empty vector, wild type HERC5, HERC5-ΔRLD or HERC5-C994A. Forty-eight hours after transfection, infectious virus released into the supernatant was measured using an infectious HIV-1 release assay (Figure 1D). The expression of HERC5 and HERC5-C994A significantly reduced the amount of infectious virus released into the supernatant compared to the control cells (*P* < 0.0001, student’s paired t test). In contrast, HERC5-ΔRLD failed to inhibit infectious virus release compared to the control cells (*P* > 0.05, student’s paired t test). Quantitative Western blot analysis of viral particles released into the supernatant and of producer cell lysates revealed that inhibition of HIV-1 particle production by HERC5 and HERC5-C994A resulted in a substantial reduction in total intracellular Gag protein and in HIV-1 particles released into the supernatant (Figure 1E and F). Cells expressing HERC5-ΔRLD produced similar levels of intracellular Gag protein as the empty vector control cells; however, these cells did not exhibit a full rescue of released HIV-1 particles into the supernatant.

Notably, HERC5-C994A inhibited infectious HIV-1 particle production significantly better than wild type HERC5and yielded substantially less intracellular Gag protein compared to wild type HERC5 and the control cells (*P* < 0.01, student’s unpaired t test) (Figure 1E and F). This enhanced activity correlated with the loss of slowly migrating species of HERC5 protein (Figure 1G). We used a standard ISGylation assay to determine if these slowly migrating forms of HERC5 represented ISGylated HERC5 protein [1]. To enhance detection of ISGylated species, wildtype HERC5 was expressed in the presence of the ISG15 conjugation system consisting of E1 activating enzyme Ube1L, E2 conjugating enzyme UbcH8 and ISG15. As shown in Figure 1H, slowly migrating forms of HERC5 were observed. When wildtype HERC5 was expressed in the presence of the ISG15-specific deconjugating enzyme Ubp43 [32], substantially less slowly migrating forms of HERC5 were observed. Cells expressing HERC5-C994A, which is defective for ISGylation, also did not yield slowly migrating forms of ISGylated HERC5-C994A protein. These data indicate that the slowly migrating forms of HERC5 are modified with ISG15.

### Endogenously expressed HERC5 inhibits intracellular Gag protein production

**Figure 2:**
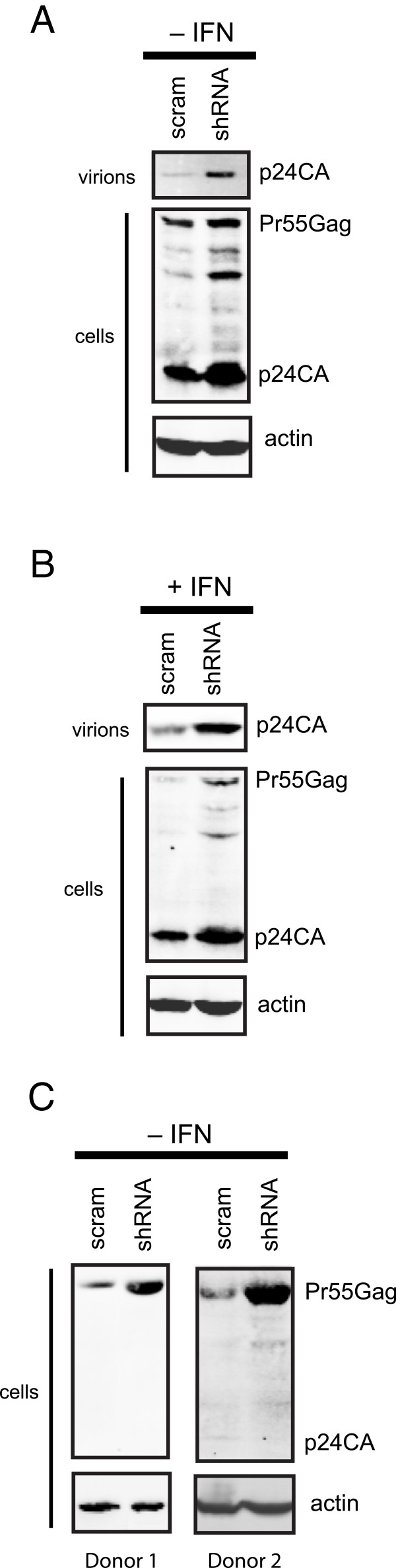
**Endogenous HERC5 inhibits intracellular Gag protein production. A**, 293T cells were co-transfected with pR9 and either pScram or pHERC5_shRNA_. Forty-eight hours after transfection, cell lysates were subjected to Western blotting using anti-p24CA or anti-β-actin as a loading control. **B**, Cells were transfected as in part A for 24 hours and then treated with 500 units/ml of IFN-β. Sixteen hours after IFN-β treatment, cell lysates were subjected to Western blotting using anti-p24CA or anti-β-actin as a loading control. Data shown in panels A and B are representative of at least three independent experiments. **C**, Human monocyte-derived macrophages from two different donors were transfected as in panel A. Seventy-two hours after transfection, cell lysates were subjected to Western blotting using anti-p24CA or anti-β-actin as a loading control.

To assess whether endogenously-expressed HERC5 inhibits intracellular Gag protein expression from infectious HIV-1, we knocked down endogenous HERC5 RNA using short hairpin RNA (shRNA) and examined the influence of reduced HERC5 expression on intracellular Gag protein production, compared with cells expressing scrambled shRNA. Western blot analysis of cell lysates from transfected 293T cells, before or after treatment with IFN-β, revealed that HERC5 shRNA-expressing cells exhibited substantially more intracellular Gag protein than cells expressing scrambled shRNA (Figure 2A and B). A similar effect was observed in primary human macrophages from two different donors (Figure 2C). Cells expressing HERC5 shRNA exhibited an average of 3.2-fold less HERC5 RNA than control cells expressing scrambled shRNA, as determined by quantitative reverse transcription polymerase chain reaction (qPCR). As a control for specificity, we similarly measured the effect of the scrambled and HERC5 shRNA on HERC3 RNA levels and found them to be equivalent. Together, these data suggest that endogenously expressed HERC5 provides a significant barrier to intracellular Gag protein production with and without IFN pre-treatment.

### HERC5 inhibits nuclear export of HIV-1 genomic RNA

**Figure 3:**
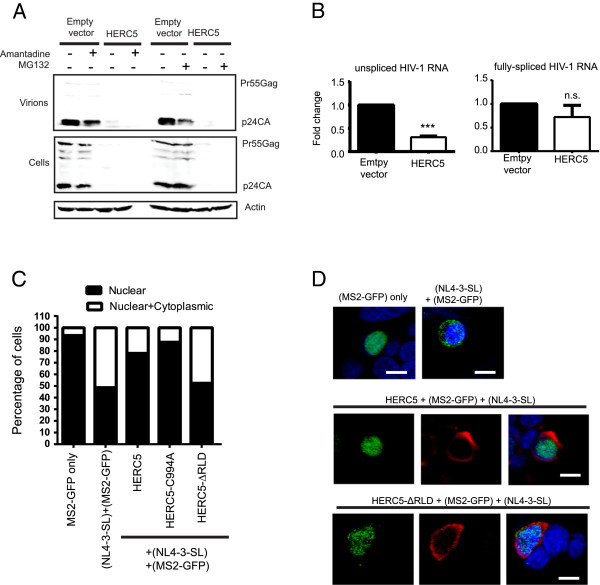
**HERC5 inhibits nuclear export of HIV-1 genomic RNA. A,** 293T cells were co-transfected with pR9 and either empty vector or pHERC5. Forty hours post-transfection, cells were treated with the proteasomal inhibitor MG132 (20 μM) or the lysosomal inhibitor amantidine (1.5 mM) for 16 hours. Virus released into the supernatant or total cell lysates were subjected to quantitative Western blot analysis using anti-p24CA and anti-β actin as a loading control. **B**, Cells were transfected as in (A). Forty-eight hours after transfection, total RNA was extracted and reverse transcribed into cDNA from whole cell lysates or from the cytoplasmic fraction only. Quantitative PCR was performed on each fraction using primers specific to unspliced HIV-1 genomic RNA (e.g. Gag), fully spliced RNA (e.g. Rev), total HIV-1 RNA (e.g. LTR), β-actin (loading control) or GFP (transfection control). The proportion of unspliced or fully-spliced HIV-1 RNA in the cytoplasmic fraction compared to total amount of HIV-1 RNA (nuclear plus cytoplasmic) was determined for control cells and cells expressing HERC5. Fold-change in copy number relative to control cells is shown. Data shown represents the average (+/- SEM) from six independent experiments. ***P = 0.0003; not significant (n.s.) *P* > 0.05 (student’s paired t test). **C** and **D**, HeLa cells were co-transfected with plasmids encoding MS2-GFP alone, MS2-GFP and NL4-3-SL, or MS2-GFP + NL4-3-SL and either flag-tagged HERC5, HERC5-C994A or HERC5-∆RLD. Forty-eight hours post-transfection, cells were fixed, stained with anti-flag and DAPI and imaged using fluorescence confocal microscopy. MS2-GFP localization was assessed in each cell and categorized according to localization in the nucleus only or both the nucleus and cytoplasm (C). Results shown are from at least three independent experiments (n = 331). Representative images of the predominant phenotypes are shown (D). Blue, nucleus; green, MS2-GFP; red, flag-tagged HERC5. Scale bars = 10 μm.

To investigate the mechanism by which HERC5 inhibits intracellular Gag protein production, we asked if HERC5 induced the degradation of intracellular Gag protein. 293T cells were co-transfected with plasmids encoding replication-competent HIV-1 and HERC5 and then treated with the proteasome inhibitor MG132 or the lysosome enzyme inhibitor amantidine. Western blot analysis of cell lysates revealed that MG132 or amantidine treatment did not rescue levels of intracellular Gag protein (Figure 3A). This finding suggests that HERC5 does not induce Gag protein degradation. MG132 and amantidine treatment also did not rescue the release of extracellular HIV-1 particles into the cell supernatant. MG132-treated control cells exhibited a reduction in the production of extracellular virus, as previously reported [33]. Similar results were obtained using U2OS cells (data not shown).

Since Gag protein is expressed from unspliced HIV-1 genomic RNA in the cytoplasm, we asked if HERC5 interfered with the nuclear export of unspliced HIV-1 RNA. 293T cells were co-transfected with plasmids encoding full-length replication-competent HIV-1 (pR9) and either empty vector or HERC5. A plasmid encoding green fluorescent protein (GFP) was also co-transfected to serve as a transfection control. Total RNA was harvested from total cell extract or cytoplasmic extract only and subjected to qPCR with primers specific to either unspliced HIV-1 genomic RNA (e.g. Gag), fully spliced RNA (e.g. Rev), total HIV-1 RNA (e.g. LTR), β-actin (loading control) or GFP (transfection control). Cells expressing HERC5 exhibited a 2.7 to 4.2-fold reduction in the amount of HIV-1 genomic RNA exported to the cytoplasm compared to the control cells (*P* =0.0003, student’s paired t test). In contrast, no significant difference was observed in the export of fully-spliced HIV-1 Rev transcripts (Figure 3B).

To further investigate the effect of HERC5 on the localization of unspliced HIV-1 RNA, we utilized an established assay involving the bacteriophage MS2 coat protein to determine the localization of HIV-1 genomic RNA. HIV-1 NL4-3 genomic RNA was tagged with 24 copies of the MS2 binding RNA stem loops (NL4-3-SL). These stem loop structures bind with high affinity and specificity to a fusion protein consisting of the bacteriophage MS2 coat protein and GFP (MS2-GFP). The MS2 RNA stem loops were inserted such that unspliced full-length genomic RNA would be labelled with MS2-GFP, as previously described [34]. MS2-GFP contains a nuclear localization signal sequence that targets the fusion protein to the nucleus; however, MS2-GFP can shuttle to the cytoplasm when bound to cargo destined for the cytoplasm.

MS2-GFP expressed alone localized exclusively in the nucleus as expected (Figures 3C, D and Additional file 1: Figure S1). When the MS2-GFP signal intensity was increased, no MS2-GFP signal was observed in the cytoplasm (Additional file 1: Figure S1B). When MS2-GFP was co-expressed with NL4-3-SL, MS2-GFP localized in both the nucleus and cytoplasm as expected. However, in the presence of HERC5 or HERC5-C994A, MS2-GFP localized predominantly in the nucleus (Figure 3C and D). Similar to the MS2-GFP only control, no MS2-GFP signal was observed in the cytoplasm after the signal intensity was increased (Additional file 1: Figure S1B). In contrast, MS2-GFP localized in both the nucleus and cytoplasm in the presence of HERC5-ΔRLD, indicating that the HERC5 RCC1-like domain is required for inhibiting nuclear export of HIV-1 genomic RNA (Figure 3C and D). Taken together, these data demonstrate that HERC5 inhibits nuclear export of unspliced HIV-1 RNA.

### HERC5 inhibits nuclear export of Rev/RRE-dependent HIV-1 RNA

In eukaryotic cells, intron-containing messages are normally retained in the nucleus, whereas completely spliced messages are allowed to exit into the cytoplasm. HIV-1 overcomes this checkpoint in cells through expression of the HIV-1 regulatory protein Rev. Rev promotes nuclear export of intron-containing HIV-1 mRNAs by binding to a specific *cis*-acting element called the rev-response element (RRE), located within the HIV-1 intron [35-39]. Rev binds to the CRM1/RanGTP complex and translocates through the nuclear pore complex to the cytoplasm via the CRM1-dependent nuclear export pathway. The constitutive transport element (CTE) from Mason-pfizer monkey virus (MPMV) is a structured RNA element that also functions in *cis,* but it does not require co-expression of a viral Rev-like protein for the nuclear export of intron-containing RNA. Instead, CTE-containing RNA recruits the NXF1/NXT1 proteins, which direct nuclear export of the RNA via the NXF1-dependent pathway and is independent of RanGTP [40-42].

**Figure 4:**
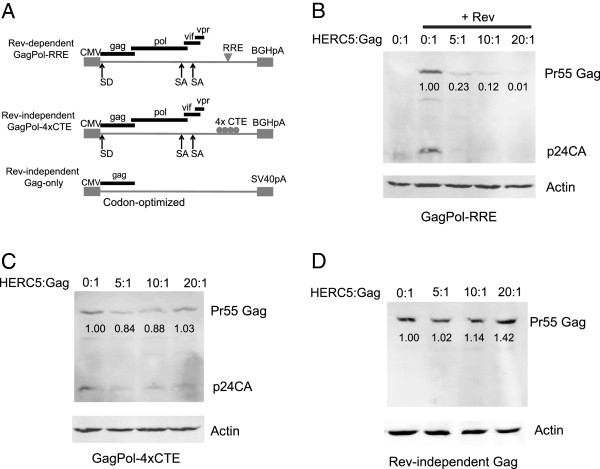
**HERC5 targets Rev/RRE function. A**, Schematic depicting the different Gag constructs used in the experiment. 293T cells were co-transfected with increasing concentrations of plasmids encoding HERC5 and either Rev-dependent GagPol (containing RRE) +/- Rev **(B)**, Rev-independent GagPol (containing 4xCTE in place of the RRE) **(C)**, or Rev-independent Gag-only (codon-optimized) construct **(D)**. Total DNA transfected was kept equal with empty vector plasmid. Gag levels within the cell lysates were analyzed by quantitative Western blotting using anti-p24CA and anti-β-actin as a loading control. Numbers below the bands represent densitometric quantification of the indicated bands relative to the empty vector control after normalization to β-actin levels.

To determine if HERC5-mediated inhibition of HIV-1 RNA nuclear export was Rev/RRE-dependent, we tested the ability of HERC5 to inhibit Gag expression from Rev-dependent (e.g. GagPol-RRE) and Rev-independent (e.g. GagPol-4xCTE and codon-optimized Gag-only) constructs, as previously described (Figure 4A) [43]. 293T cells were co-transfected with increasing concentrations of plasmids encoding HERC5, with or without pGagPol-RRE, pGagPol-4xCTE or pGag. As shown in Figure 4B, HERC5 potently inhibited Gag expression from the GagPol-RRE construct. In contrast, a modest reduction in Gag expression was observed from the GagPol-4xCTE construct (Figure 4C). No reduction in Gag expression was observed from the RRE/CTE-independent Gag-only construct (Figure 4D).

### HERC5 targets RanGTP and alters Rev localization

**Figure 5:**
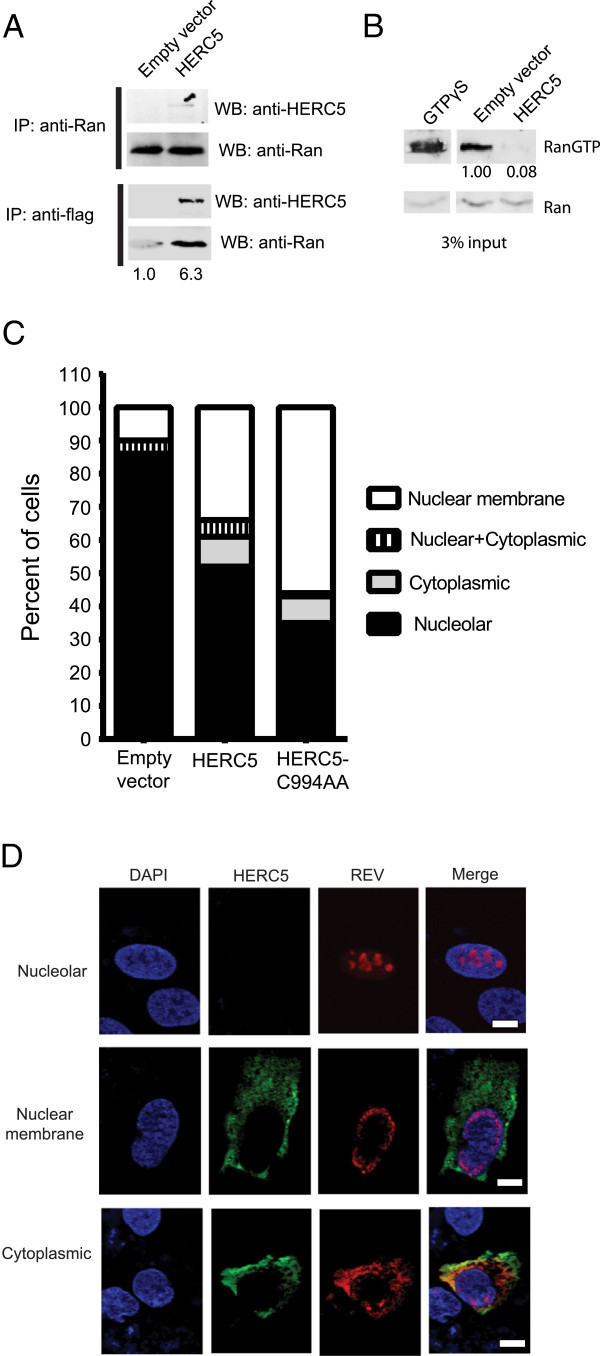
**HERC5 interacts with Ran and reduces RanGTP levels and/or its binding with RanBP1. A**, 293T cells were co-transfected with either empty vector or a plasmid encoding flag-tagged HERC5. Forty-eight hours after transfection, cells were lysed under non-denaturing conditions and subjected to immunoprecipitation using anti-Ran or anti-flag. Precipitated proteins were separated by SDS-PAGE and subjected to Western blotting using anti-HERC5 or anti-Ran. Numbers below the lower blot represent the densitometric quantification of the non-specific band in the empty vector control and Ran in the HERC5-expressing cells. **B**, U2OS cells were transiently-transfected with empty vector or a plasmid encoding HERC5. Forty-eight hours after transfection, total cell lysate was mixed with RanBP1-bound agarose beads to selectively isolate and pull-down RanGTP. As a control, cell lysate was incubated with non-hydrolyzable GTPγS prior to incubation with the agarose beads. The eluate, including input lysate (3%), were separated by SDS-PAGE gel and subjected to quantitative Western blotting using anti-Ran. Note, bands shown in the input samples represents total Ran protein and does not distinguish RanGTP or RanGDP. The blot shown is representative of three independent experiments. Numbers represent the densitometric quantification of RanGTP after normalization to Ran levels. **C**, U2OS cells were co-transfected with plasmids encoding HIV-1 Rev and either flag-tagged HERC5 or HERC5-C994A. Forty-eight hours post-transfection, cells were fixed, stained with anti-flag and/or anti-Rev and DAPI and imaged using fluorescence confocal microscopy. Rev localization was assessed in each cell and categorized as either: near the nuclear membrane, in both the nucleus and cytoplasm, in the cytoplasm only, or nucleolar. Results shown are from at least three independent experiments (n = 500). **D**, Representative images of the predominant phenotypes. Blue, nucleus; green, flag-tagged HERC5; red, Rev. Scale bars = 10 μm.

RCC1 is localized in the nucleus and stimulates the conversion of RanGDP into RanGTP. A steep gradient of RanGTP between the nucleus and cytoplasm is essential for Crm1-dependent nuclear export, but not the NXF1-dependent export [31]. To determine if HERC5 interacts with Ran, cells expressing or not expressing flag-tagged HERC5 were lysed under non-denaturing conditions and subjected to co-immunoprecipitation using anti-Ran or anti-flag. Western blot analysis of the precipitated proteins revealed that Ran and HERC5 co-precipitated (Figure 5A). We then asked if HERC5 expression affected intracellular RanGTP levels. Due to the lack of a specific antibody that distinguishes RanGTP from RanGDP, we utilized a RanGTP pull-down assay involving Ran binding protein 1 (RanBP1)-coated agarose beads. RanBP1 binds specifically to RanGTP and not RanGDP. Control cells treated with non-hydrolyzable GTPγS, transfected with empty vector, or transfected with pHERC5 were lysed and mixed with RanBP1-coated beads. RanGTP protein eluent was measured using quantitative Western blotting with anti-Ran. Control cells treated with GTPγS and empty vector control cells readily pulled down RanGTP (Figure 5B). In stark contrast, substantially less RanGTP was pulled down in cells expressing HERC5, indicating that HERC5 reduced intracellular levels of RanGTP or interfered with the interaction between RanGTP and RanBP1.

Since nuclear-cytoplasmic shuttling of HIV-1 Rev is critically dependent on RanGTP, we asked if the effect of HERC5 on RanGTP correlated with aberrant Rev localization. U2OS cells, which do not express endogenous HERC5, were co-transfected with plasmids encoding flag-tagged HERC5 and HIV-1 Rev and imaged by confocal immunofluorescence microscopy. Quantitative analysis of cells co-expressing HERC5 and Rev revealed that 34% of cells expressing HERC5 and 56% of cells expressing HERC5-C994A exhibited Rev localization at or near the nuclear membrane compared to 10% in the control cells (*P* = 0.0004 and *P* < 0.0001 respectively, Fisher’s exact test) (Figure 5C and D). Nine percent of cells expressing HERC5 and 8% of cells expressing HERC5-C994A exhibited Rev localization in the cytoplasm only compared to 0% in the control cells (*P* = 0.0032 and *P* = 0.0032 respectively, Fisher’s exact test) (Figure 5C and D). These data indicate that HERC5 expression significantly alters the subcellular localization of Rev.

### Positive selection is operating on HERC5

The antiviral activities of HERC5 towards evolutionarily diverse viruses, together with the realization that HERC5 orthologs exist in evolutionarily diverse mammals, identifies HERC5 as a candidate host factor that has likely experienced genetic conflict with viruses during mammalian evolution. Therefore, we asked if positive evolutionary selection is operating on HERC5. The computer software Selecton (Server for the Identification of Site-Specific Positive Selection & Purifying Selection) combines the implementation of state-of-the-art methods for detecting positive evolutionary selection [44,45]. Selecton has been shown to successfully detect site-specific selection forces on the retroviral restriction factor TRIM5α, a protein that has recently been shown to have undergone positive selection during the course of primate evolution [44,46,47]. Selecton analysis also enabled the detection of positively selected regions that correlated with the previously identified species-specificity determinants of TRIM5α [44].

**Figure 6:**
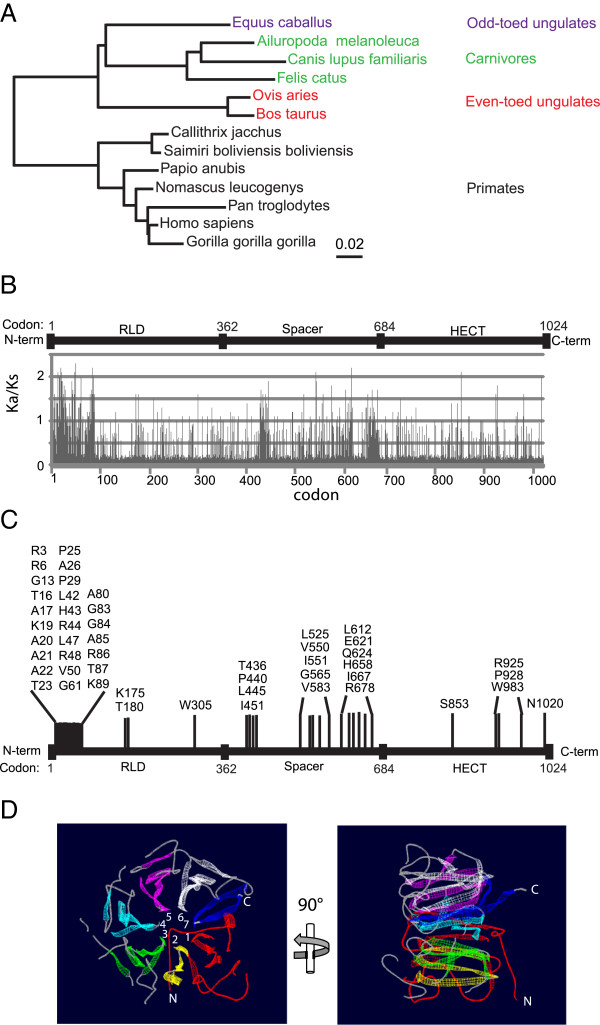
**Positive evolutionary selection analysis of HERC5. A**, Neighbor-joining phylogenetic tree for progressive alignment of 13 different HERC5 species using constraint-based alignment tool (COBALT) for multiple protein sequences (Additional file 1: Figure S4). Branch lengths are proportional to the amount of inferred evolutionary changes. **B** and **C**, Selecton analysis for positive selection was performed using HERC5 sequences from human, chimpanzee, gorilla, marmoset, baboon, squirrel monkey, gibbon, horse, panda, sheep, cow, dog and cat. Evolutionary analysis for positive selection in HERC5 using various models of evolution where M8 and MEC allow for sites to evolve under positive selection and M7 and M8a models do not. **(B)** A plot of the Ka/Ks ratio at each codon in an alignment of HERC5 coding sequences is shown. Codons with Ka/Ks ratios >1 indicate positive selection, =1 neutral selection and <1 purifying selection. **C**, A plot showing the results of a Bayesian analysis approach to identify sites where Ka/Ks >1, mapped to the different HERC5 domains. Shown are the sites where Ka/Ks >1.5 and the 95% confidence interval is larger than 1; hence considered statistically significant. **D**, The HERC5 RLD was modeled using SWISS-MODEL (Swiss Institute of Bioinformatics: http://swissmodel.expasy.org/) and visualized and colored using DeepView/Swiss-PDBViewer, v4.0.1. The region corresponding to amino acids 2–103 is colored red. All other colors are arbitrary and used to highlight the different blades of the β-propeller structure. The inset numbers identify the different blades. “N” and “C” denote the amino- and carboxyl-termini respectively.

We used a similar Selecton analysis to test for positive selection on HERC5 using 13 evolutionarily diverse HERC5 sequences as input sequences (Figure 6A and Additional file 1: Figure S2). HERC5 evolution in mammals was evaluated under several standard models of sequence evolution as implemented in the Selecton program. This comprised two nested pairs of models (M8a and M8; and M7 and M8), in which the first model of each pair is nested in the second model. The M8 model, but not the M8a or M7 models, allows sites to evolve under positive selection. A non-nested pair (M8a and MEC) model comparison was also performed. The MEC model differs from the other models in that it takes into account the differences between amino acid replacement rates [44]. The nested models were compared using the likelihood ratio test. In each case, allowing sites to evolve under positive selection (M8) gave a significantly better fit to the HERC5 sequence data than the corresponding model without positive selection (M8a and M7) (Figure 6B). The MEC model, which allows for positive selection, was compared with the M8a null model, which does not allow for positive selection. Comparison of the AIC_c_ scores (M8a: 28432; MEC: 28016) revealed that the MEC model fits the HERC5 data better than the M8a model (Additional file 1: Table S1). The results of the MEC analysis were projected by Selecton onto the primary sequence of human HERC5 (Figures 6B, C and Additional file 1: Figure S3). The results show that positive selection is operating on HERC5 and that several codons situated in the RCC1-like domain, the spacer region and the HECT domain exhibit exceptionally high probabilities of having evolved under positive selection. Notably, 27 out of 50 of these codons cluster within the first 100 amino acids of the amino terminus of the RCC1-like domain, encompassing blade 1 and part of blade 2 of its predicted 7-bladed β-propeller structure (Figure 6D).

**Figure 7:**
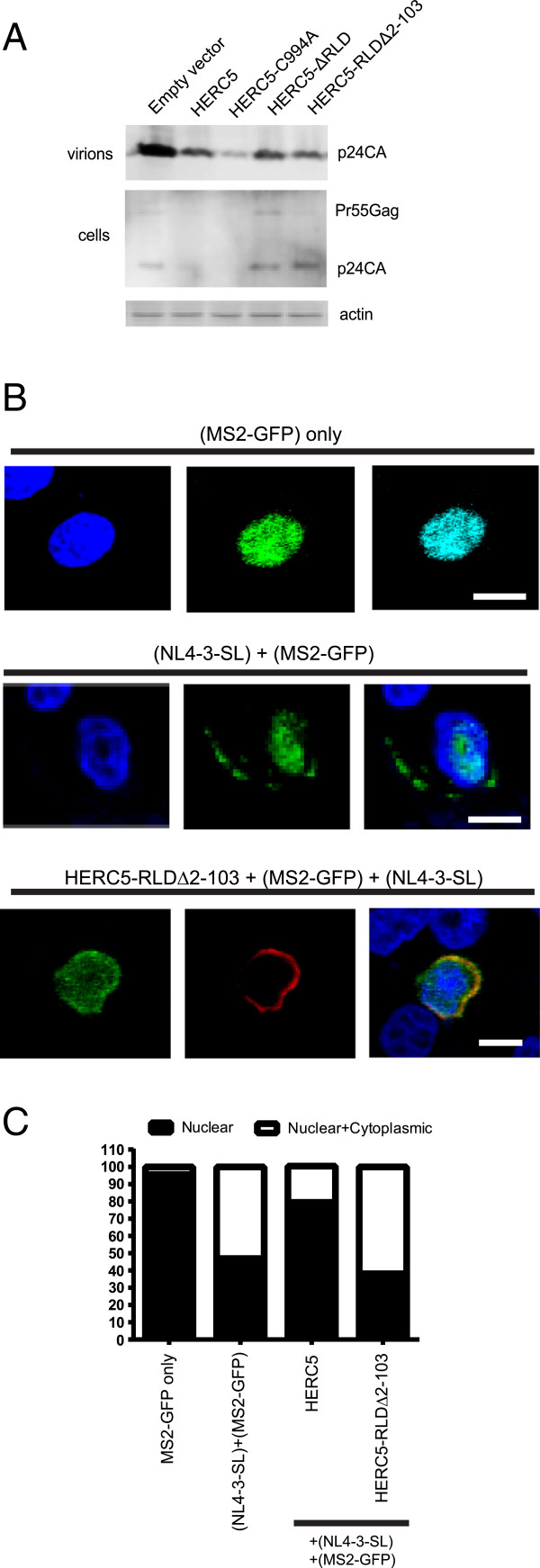
**Amino acids 2–103 of HERC5 are required for inhibiting nuclear export of HIV-1 genomic RNA. A**, Western blot analysis of 293T cells co-transfected with pR9 and either empty plasmid or a plasmid encoding HERC5, HERC5-C994A, HERC5-∆RLD and HERC5-RLD∆2-103. Forty-eight hours after transfection, HIV-1 virions released into the supernatant and Gag levels within the cell lysates were analyzed by quantitative Western blotting using anti-p24CA or anti-β-actin as a loading control. **B**, HeLa cells were co-transfected with plasmids encoding MS2-GFP alone, MS2-GFP and NL4-3-SL, or MS2-GFP + NL4-3-SL and either flag-tagged HERC5, HERC5-C994A, HERC5-∆RLD or HERC5-RLD∆2-103. Forty-eight hours post-transfection, cells were fixed, stained with anti-flag and DAPI and imaged using fluorescence confocal microscopy. Representative images of the predominant phenotypes are shown. Blue, nucleus; green MS2-GFP; red, flag-tagged HERC5. Scale bars = 10 μm. **C**, MS2-GFP localization was assessed in each cell and categorized according to localization in the nucleus only or both the nucleus and cytoplasm. The results of the phenotypic quantification are shown and were obtained from at least three independent experiments (n = 235).

Since there was a marked enrichment in positively selected sites within blades 1 and 2, we generated a HERC5 mutant lacking amino acids 2–103 (HERC5-RLDΔ2-103) and tested whether these blades contributed to the HERC5-mediated inhibition of HIV-1 particle production. Similar to our observations with HERC5-ΔRLD, HERC5-RLDΔ2-103 produced similar levels of intracellular Gag protein as the empty vector control cells and did not exhibit a full rescue of released HIV-1 particles into the supernatant (Figure 7A). Furthermore, HERC5-RLDΔ2-103 failed to inhibit nuclear export of HIV-1 genomic RNA compared to wild type HERC5 (Figure 7B and C). These findings indicate that blades 1 and 2 are required for the HERC5-mediated inhibition of nuclear export.

## Discussion

Previously, we showed that HERC5 inhibits an early stage of HIV-1 Gag assembly at the plasma membrane by a mechanism correlating with the modification of Pr55Gag with ISG15 [1]. Here we demonstrate that HERC5 inhibits HIV-1 particle production by a second distinct mechanism targeting Rev/RRE function. A region of the RCC1-like domain of HERC5 was required for this inhibition, which is also evolving under strong positive selection. Although deleting the RCC1-like domain of HERC5 rescued inhibition of Rev/RRE function, HERC5-ΔRLD inhibited HIV-1 particle release at levels comparable to wild type HERC5. HERC5-ΔRLD has been shown to possess some E3 ligase activity for ISG15 conjugation; therefore, it is likely that this E3 ligase activity contributed to inhibition of particle release via ISGylation of HIV-1 Gag [19,48]. However, the infectivity of HIV-1 particles released from cells expressing HERC5-ΔRLD did not differ from those released from the control cells. By inhibiting the nuclear export of HIV-1 genomic RNA, the RCC1-like domain may also promote the release of non-infectious HIV-1 particles (i.e. particles lacking genomic RNA). We also showed that HERC5-C994A was able to inhibit HIV-1 particle production better than wild type HERC5. This potent inhibition correlated with the loss of ISGylated forms of HERC5. Given that HERC5 modifies itself with ISG15, it is possible that auto-ISGylation negatively regulates HERC5 antiviral activity. This auto-regulation may represent a mechanism by which HERC5 maintains tight control over its E3 ligase-independent activity, particularly during periods of high-expression such as after induction by IFN. A similar finding was previously observed for the E3 ligase TRIM25 where auto-ISGylation negatively regulated its activity for conjugating ISG15 to 14-3-3sigma [49].

HERC5 expression is significantly up-regulated in cells after exposure to IFN [18,19]. We showed here that knocking down HERC5 in a background of IFN exposure substantially increased intracellular HIV-1 Gag production after 48 hours, indicating that HERC5 is an important mediator of the IFN response towards HIV-1. Knocking down endogenous levels of HERC5 in primary macrophages, in the absence of exogenous IFN, also resulted in a substantial increase in HIV-1 particle production. This finding suggests that endogenous levels of HERC5 may serve to limit, but not fully restrict, HIV-1 particle production in the absence of IFN. As HERC5 levels increase, such as after exposure to IFN, HERC5 may be more able to restrict HIV-1 particle production. This finding contrasts our previous finding that knockdown of HERC5 did not exhibit a substantial effect on the intracellular production of Pr55Gag from full-length replication-competent HIV-1 [1]. A likely explanation for the difference is that the data in the present study was obtained 48 hours post-transfection, compared to 24 hours in our previous study. Another contributing factor could be that the level of HERC5 knockdown achieved in the present study was higher than our previous study (3.2-fold versus 2.3-fold respectively). In both studies, HERC5 had no substantial effect on intracellular Pr55Gag levels when expressed from a Rev-independent Gag-only construct.

Most eukaryote messenger RNAs undergo splicing to remove introns before they are exported to the cytoplasm via the NXF1/NXT1-dependent nuclear export pathway [50-53]. However, the expression of HIV-1 genes is a notable exception. Unspliced and singly-spliced HIV-1 RNA must be exported to the cytoplasm before they are fully-spliced by host machinery in the nucleus [54-57]. These incompletely-spliced RNAs are essential for steps such as incorporation of full-length genomes into new virions and for expression of Gag, Gag-Pol, Env, Vif, Vpr and Vpu proteins. Nuclear export of incompletely-spliced HIV-1 RNAs occurs when the Rev/RRE complex recruits the dimeric complex of Crm1/RanGTP before translocating through the nuclear pore to the cytoplasm via the Crm1/RanGTP-dependent nuclear export pathway. Once in the cytoplasm, the complex dissociates and RanGTP is converted to RanGDP with the help of RanBP1 and RanGAP1, which then shuttles back into the nucleus for another round of export [35-39,58].

We showed that HERC5 interacts with Ran and substantially reduces intracellular levels of RanGTP and/or inhibits the association of RanGTP with RanBP1. Cells require a high concentration (>1000-fold) of RanGTP in the nucleus, which is believed to provide directionality for nuclear export to the cytoplasm [31]. Perturbing this nuclear:cytoplasmic RanGTP gradient by either reducing total RanGTP levels or increasing the cytoplasmic RanGTP level by interfering with the interaction between RanGTP and RanBP1, halts nuclear export of Crm1/RanGTP-dependent cargo. Consistent with this idea, we observed altered localization of Rev protein and Rev/RRE-dependent RNA in the presence of HERC5. With the ability of HERC5 to interact with Ran, it is possible that HERC5 binds and sequesters Ran in the cytoplasm. This activity would interfere with the shuttling of RanGDP into the nucleus, thereby interfering with the production of RanGTP in the nucleus. Another possibility is that HERC5 stimulates guanine nucleotide release from Ran in the cytoplasm. It was previously shown that the related RCC1-like domain 1 of human HERC1 stimulates GDP release from the small GTPase proteins ARF1/6 and Rab, but not from Ran [59,60]. Therefore, it is plausible that the HERC5 RCC1-like domain performs a similar function on Ran, with which it interacts. Further experiments are needed to further dissect this mechanism.

*HERC5* orthologs have been identified in a variety of evolutionarily diverse mammals spanning >75 million years of evolution. Genetic conflict arising from the co-evolution of hosts and pathogens can lead to rapid selection of amino acid substitutions that alter amino acid composition of the host factors and their pathogen antagonists, thereby conferring an evolutionary advantage to the host or the pathogen [61,62]. This process of positive selection is not a common phenomenon and is typically not apparent in most examined datasets [63,64]. However, recent evolutionary studies on host antiviral factors have shown that they are rapidly evolving genes due to genetic conflict between hosts and pathogens [65]. Several of these host factors such as apolipoprotein B mRNA-editing enzyme catalytic polypeptide 3G (APOBEC3G) [66]; tripartite motif protein 5 alpha (TRIM5α) [46]; bone marrow stromal antigen 2 (BST2)/tetherin [67,68]; sterile alpha motif (SAM) domain and histidine/aspartic acid domain (HD)-containing protein 1 (SAMHD1) [69]; MxA [70] contain genetic ‘signatures’ of positive selection. Positively selected residues have been shown to play key functional roles in the antiviral activities of these proteins.

We demonstrated here that *HERC5* also contains genetic signatures of strong positive selection. Twenty-seven of 50 codons predicted to be evolving under strong positive selection in HERC5 map to blades 1 and 2 of the predicted β-propeller structure of the RCC1-like domain. This finding identifies blades 1 and 2 as a functionally important region of HERC5 and may represent a highly dynamic interface with viral antagonists. Fifteen of 50 codons predicted to be evolving under strong positive selection map to the spacer region of HERC5. The high proportion of amino acids predicted to be evolving under purifying selection in blades 3–7 of the RCC1-like domain and the majority of the HECT domain indicates that purifying selection is playing an important role in maintaining the long-term stability of these domains. These two domains are highly conserved in evolutionarily diverse mammals, suggesting they play fundamental roles in biology. Indeed, the HECT domain of HERC5 confers its E3 ligase activity and HERC5 has been shown to be the main cellular E3 ligase for host ISGylation.

## Conclusions

Here we have demonstrated that HERC5 possesses a second distinct mechanism by which it blocks HIV-1 particle production. By being able to inhibit both nuclear export of incompletely-spliced HIV-1 RNA and an early step in HIV-1 Gag assembly at the plasma membrane, HERC5 represents a significant challenge for HIV-1 replication. The work presented here contributes to the growing body of evidence that HERC5 is a novel host restriction factor. Currently, HERC5 satisfies 3 of the 4 hallmarks of restriction factors: HERC5 exhibits strong ‘signatures’ of positive selection, is up-regulated by IFNβ and virus infection, and has antiviral activity as its major biological function. A direct viral antagonist of HERC5 is yet to be identified; although antagonists to HERC5 function (e.g. ISGylation) have been identified from several evolutionarily diverse viruses [71-78]. It will be interesting to discover the evolutionary pressures that drive positive selection in HERC5 and how HIV-1 and/or other viruses circumvent HERC5 activity *in vivo*.

## Methods

### Ethics statement

Informed consent was obtained from all subjects according to an ethics protocol approved by The University of Western Ontario Research Ethics Board for Health Sciences Research Involving Human Subjects (HSREB).

### Cells and cell lines

All cell lines were obtained from American Type Culture Collection unless otherwise stated. Cells were maintained in standard growth medium (Dulbecco’s Modified Eagle’s Medium (DMEM)), supplemented with 10% heat-inactivated Fetal Bovine Serum (FBS), 100 U/ml Penicillin and 100 μg/ml Streptomycin) at 37°C with 5% CO_2_. Human primary monocyte-derived macrophages were generated from peripheral blood mononuclear cells (PBMCs) of healthy volunteer donors. PBMCs were isolated by density gradient centrifugation using Histopaque-1077 (Sigma). Monocytes were purified using a CD14+ cell isolation kit from Miltenyi Biotec. The purity of CD14+ cells was >90% as determined by flow cytometric analysis using anti-human CD14 PE-Cyanine7 (eBiosciences). Monocytes were cultured for 6 days in RPMI-1640 supplemented with 10% FBS and 50 ng/ml recombinant human granulocyte-macrophage colony stimulating factor (GM-CSF) (Peprotech) as previously described [79]. The following reagents were obtained through the NIH AIDS Research and Reference Reagent Program, Division of AIDS, NIAID, NIH: (GHOST (3) R3/X4/R5; Cat. 3943) from Dr. Vineet N. KewalRamani and Dr. Dan R. Littman [80].

### Plasmids, transfections and antibodies

Plasmids: pR9, pUbe1L, pUbcH8, myc-tagged ISG15 (pMyc-ISG15), flag-tagged HERC5 (pHERC5) and flag-tagged HERC5-C994A (pHERC5-C994A) were described previously [1]. The promoterless empty vector plasmid pGL3 was purchased from Promega, p3xFLAG from Sigma, and pUbp43 from Thermo Scientific. Plasmids encoding HERC5-ΔRLD, and HERC5-RLDΔ2-103 were generated using standard domain deletion mutagenesis using the QuikChange® Lightning Site-Directed Mutagenesis Kit (Stratagene) according to manufacturer’s instructions. Primer pairs used in the reactions are as follows: HERC5-ΔRLD = forward- 5′ GAC ATG GAG CGC CGC AGC ATG ATT GCT GGA GGG AAT CAA AGC ATT TTG CTC TGG 3′ and reverse 5′ GCT TTG ATT CCC TCC AGC AAT CAT GCT GCG GCG CTC CAT GTC GTC 3′; HERC5-RLDΔ2-103 = forward 5′ GAC ATG GAG CGC CGC AGC CAG GGA GCC GAA CAC ATG CTG 3′ and reverse 5′ GTG TTC GGC TCC CTG GCT GCG GCG CTC CAT GTC GT 3′. pMS2-GFP was obtained from Addgene (cat.# 27121). pNL4-3-SL was kindly provided by Dr. Hu (National Cancer Institute, Frederick, Maryland, USA). pGagPol-RRE and pGagPol-4xCTE were provided by Dr. M. Malim (King’s College London). pScram was described previously [1]. pHERC5_shRNA_ (cat. #RHS4533-NM_016323, TRCN0000004171) was obtained from Thermo Scientific (Open Biosystems). Transfections: plasmid transfections were performed using standard calcium phosphate transfection or Lipofectamine 2000 (Invitrogen). Transfection of primary human macrophages was performed using GenJet™ In Vitro DNA Transfection Reagent for Primary Macrophages (FroggaBio). Co-transfections were performed at a ratio of 10:1 (pHERC5 construct:pR9) unless otherwise stated. The following reagents were obtained through the NIH AIDS Research and Reference Reagent Program, Division of AIDS, NIAID, NIH: HIV-1 p24 Monoclonal Antibody (183-H12-5C) from Dr. Bruce Chesebro and Kathy Wehrly [81-83]). Antibodies: anti-HERC5 was obtained from Abnova, anti-FLAG from Sigma, and anti-myc and anti-β-actin from Rockland.

### Quantification of infectious virus

Clarified supernatants containing virus particles were pelleted over a 20% sucrose cushion for 2 hours at 21,000 × g and lysed for quantitative Western blot analysis. Alternatively, the clarified supernatants were used to infect GHOST(3(3)R3/X4/R5 indicator cells. Quantification of infectious virus release using GHOST(3)R3/X4/R5 indicator cells has been described previously [80].

### Quantitative real-time PCR

Total RNA was extracted from total cell lysate or the cytoplasmic fraction only using the R&A-BLUE Total RNA Extraction kit (Frogga Bio). 3 μg of RNA was reverse transcribed to cDNA using the M-MLV reverse transcriptase and Oligo(dT) primers (Life Technologies). Prior to real-time PCR, cDNA samples were diluted 1:10 with water. Each PCR reaction consisted of 10 μl of SYBR Green Master Mix, 2 μl of Gag or Rev-specific primers (1 μl of 10 μM forward primer and 1 μl of 10 μM reverse primer), 1 μl of diluted cDNA, and water to a total volume of 20 μl. Real-time PCR was run on the Rotor-Gene 6000 real-time PCR machine (Corbett Life Science) under the following cycling conditions: 10 min at 95°C and 40 cycles of 10 sec at 95°C, 15 sec at 60°C, and 20 sec at 72°C. The Rotor-Gene 6000 series software (version 1.7) was used to determine the C_T_ for each PCR reaction.

### Western blotting and confocal microscopy

Cells were cultured in 12-well plates on 18 mm coverslips and co-transfected with pHERC5 (or pHERC5-C994A or empty vector), pUbcH8, pUbe1L, pISG15 and pGag (10:5:5.5:1 ratio respectively). Twenty-four hours post-transfection, the coverslips containing the cells were washed twice with PF buffer (1× PBS + 1% FBS), fixed for 10 minutes in 1× PBS containing 5% formaldehyde and 2% sucrose, permeabilized in 1× PBS containing 5% NP-40 and then washed twice more with PF buffer. The coverslips were incubated with primary antibodies for one hour, washed 6× with PF buffer, incubated with secondary antibodies (Alexa Fluor 546 anti-mouse or AlexaFluor 488 anti-rabbit, Invitrogen) for one hour and then washed 6× with PF buffer. Coverslips were mounted onto glass slides with ~10 μl of Vectashield mounting media with DAPI (Vector Laboratories) and then sealed with nail polish. Slides were examined using a Zeiss LSM 510 confocal fluorescence microscope and images were obtained with sequential imaging.

### Immunoprecipitation

For immunoprecipitation, cells were lysed with cold non-denaturing lysis buffer (1% (w/v) SDS, 50 mM Tris-Cl pH 7.4, 5 mM EDTA pH 8.0, 300 mM NaCl, 0.02% (w/v) sodium azide with Roche protease inhibitor) for 20 minutes. Cyanogen bromide-activated Sepharose beads (GE Health Care) were swollen in 1 mM HCl for 10 minutes followed by antibody coupling using either 5 μl of rabbit anti-p24CA or 1 μl of mouse anti-FLAG (Sigma) per 75 μl of beads in coupling buffer (0.1 M NaHCO_3_ pH 8.3 with 0.5 M NaCl) overnight at 4°C. The beads were then washed three times with coupling buffer to remove excess antibody. The beads were blocked with blocking buffer (0.1 M Tris–HCl buffer pH 8.0) for 2 hours at 4°C. The beads where then washed with 3 cycles of alternating pH (0.1 M sodium acetate pH 4 with 0.5 M NaCl; 0.1 M Tris–HCl pH 8.0 with 0.5 M NaCl). The cell lysates were added to the beads for 1 hour at 4°C. The beads were washed 8 times with non-denaturing lysis buffer and the protein was eluted with 0.5 mM NaCl.

### RanGTP pull-down assay

Forty-eight hours after transfection, the media was aspirated from a confluent 10 cm dish of 293T cells. Cells were washed twice with ice-cold 1x PBS, scraped from the dish and placed into an appropriately sized tube and kept on ice at all times. Cells were processed using the Ran Activation Assay Kit (Cell BioLabs, Inc.) according to the manufacturer’s protocol.

### Positive selection analysis

HERC5 sequences were aligned and a phylogenetic tree generated using COBALT (http://www.ncbi.nlm.nih.gov/tools/cobalt/) [84]. *HERC5* sequences were obtained from Genbank: *Homo sapiens* (“Human”) (NP_057407.2), *Pan troglodytes* (“Chimpanzee”) (XP_003310459.1), *Gorilla gorilla gorilla* (“Gorilla”) (XP_004039179.1), *Callithrix jacchus* (“Marmoset”) (XP_002745648.1), *Papio anubis* (“Baboon”) (XP_003898997.1), *Saimiri boliviensis boliviensis* (“Squirrel monkey”) (XP_003924055.1), *Nomascus leucogenys* (“Gibbon”) (XP_003265940.1), *Equus caballus* (“Horse”) (XP_001915115.2), *Ailuropoda melanoleuca* (Giant Panda”) (XP_002913645.1), *Ovis aries* (“Sheep”) (XP_004009762.1), *Bos taurus* (“Cow”) (NP_001095465.1), *Canis lupus familiaris* (“Dog”) (XP_535652.3), *Felis catus* (“Cat”) (XP_003985249.1). At least 2 independent sequences were available for human, sheep, baboon, marmoset, gibbon, squirrel monkey. The following sequences were not independently validated: cat, dog, cow, horse, sheep and giant panda. The identification of site-specific positive selection and purifying selection was generated using the Selecton Server (http://selecton.tau.ac.il/index.html) as previously described [44,45]. The HERC5 phylogenetic tree was used in the Selecton analysis. Nested pairs of models (M8a and M8; and M7 and M8) and a non-nested pair (M8a and MEC) were compared using the likelihood ratio test implemented in the Selecton program.

### Statistical analyses

GraphPad Prism v5.03 was used for all statistical analyses stated in the text. *P* values and statistical tests used are stated in the text where appropriate. *P* values less than 0.05 were deemed significant.

### Highlights

– HERC5 targets HIV-1 Rev/RRE function by a mechanism requiring the RCC1-like domain.

– HERC5 is evolving under positive evolutionary selection.

– Blades 1 and 2 of HERC5 are required for inhibiting nuclear export.

– HERC5 interacts with Ran and targets RanGTP.

## Abbreviations

IFN: Interferon; LPS: Lipopolysaccharide; ISG15: Interferon-stimulated protein, 15 kDa; HERC5: HECT domain and RCC1-like domain-containing protein 5; RCC1: Regulator of Chromosome Condensation 1-like domain; HECT: Homologous to the E6-AP Carboxyl Terminus; HIV-1: Human immunodeficiency virus type 1; shRNA: Short hairpin RNA; RT-PCR: Reverse transcription-polymerase chain reaction; RRE: Rev response element.

## Competing interests

The authors declare that they have no competing interests.

## Author’s contributions

MWW and SDB. conceived, designed and performed the experiments. JDD and SMMH assisted with the experimental design. JGT, SKT, PCC and PAS helped perform experiments. SDB and MWW wrote the paper. All authors read and approved the final manuscript.

## Supplementary Material

Additional file 1 Description of data: Table S1, Figure Legends, Figure S1, Figure S2, Figure S3. Click here for file
