# Supplementary material for: Interferon-induced HERC5 is evolving under positive selection and inhibits HIV-1 particle production by a novel mechanism targeting Rev/RRE-dependent RNA nuclear export
Source: Retrovirology. 2014 Apr 3;11:27. doi: 10.1186/1742-4690-11-27 (PMC4021598; doi:10.1186/1742-4690-11-27)
Supplement: Additional file 1 — Description of data: Table S1, Figure Legends, Figure S1, Figure S2, Figure S3. [file 1742-4690-11-27-S1.pdf]

Table S1: Analysis for positive selection in HERC5

Nested models	Log-likelihood	$2(Ln_1-Ln_0)$	Degrees of Freedom	X^2
**M8a, M8**	-14212, -14193	38	1	<0.001
**M7, M8**	-14224, -14193	62	2	<0.001

Non-nested model	Log-likelihood	AIC_c Score$^{\Delta}$: $-2*\log L+2p*(N/N-p-1)$	Parameters
**M8a, MEC**	-14212, -14003	28432, 28016	M8a: 4 free parameters<br>MEC: 5 free parameters

$^{\Delta}$ *L* represents the likelihood of the model given the data, *p* represents the number of free parameters and *N* represents the sequence length. The lower the AIC_C score, the better the fit of the model to the data, and hence the model is considered more justified.

SUPPLEMENTAL FIGURE LEGENDS

Supplemental Figure 1: HERC5 inhibits nuclear export of HIV-1 genomic RNA. A, HeLa cells were co-transfected with plasmids encoding MS2-GFP alone, MS2-GFP and NL4-3-SL, or MS2-GFP, NL4-3-SL and either flag-tagged HERC5, HERC5-C994A or HERC5-ΔRLD. Forty-eight hours post-transfection, cells were fixed, stained with anti-flag and DAPI and imaged using fluorescence confocal microscopy. **B**, Over-exposure of cells exhibiting nuclear localization of MS2-GFP. HeLa cells were co-transfected with plasmids encoding MS2-GFP and NL4-3-SL and either flag-tagged HERC5, or HERC5-C994A. Forty-eight hours post-transfection, cells were fixed, stained with anti-flag and DAPI and imaged using fluorescence confocal microscopy. Cells exhibiting nuclear localization were imaged after increased laser intensity (also see **Figure 3**).

Supplemental Figure 2: Alignment of protein sequence for HERC5 mammalian species. The constraint-based multiple alignment tool (COBALT) was used to perform a progressive multiple alignment of HERC5 amino acid sequences from human, chimpanzee, gorilla, marmoset, baboon, squirrel monkey, gibbon, horse, giant panda, sheep, cow, dog and cat. Pubmed accession numbers are as follows: *Homo sapiens* (”Human”) (NP_057407.2), *Pan troglodytes* (”Chimpanzee”)(XP_003310459.1), *Gorilla gorilla gorilla* (”Gorilla”) (XP_004039179.1), *Callithrix jacchus* (”Marmoset”) (XP_002745648.1), *Papio anubis* (”Baboon”) (XP_003898997.1), *Saimiri boliviensis boliviensis* (”Squirrel monkey”) (XP_003924055.1), *Nomascus leucogenys* (”Gibbon”) (XP_003265940.1), *Equus caballus* (”Horse”) (XP_001915115.2), *Ailuropoda melanoleuca* (Giant Panda”) (XP_002913645.1), *Ovis aries* (”Sheep”) (XP_004009762.1), *Bos taurus* (”Cow”) (NP_001095465.1), *Canis lupus familiaris* (”Dog”) (XP_535652.3), *Felis catus* (”Cat”) (XP_003985249.1) (see also **Figure 6**).

Supplemental Figure 3: Selecton results for HERC5 run on 13 HERC5 sequences with the MEC model. Positive selection is colored in shades of yellow, neutral selection in white, and purifying selection in shades of magenta. Shades of yellow (colors 1 and 2) indicate a Ka/Ks ratio > 1, white indicates a Ka/Ks ratio = 1, and shades of magenta (colors 3 through 7) indicate a Ka/Ks ratio < *1* (see also **Figure 6**).

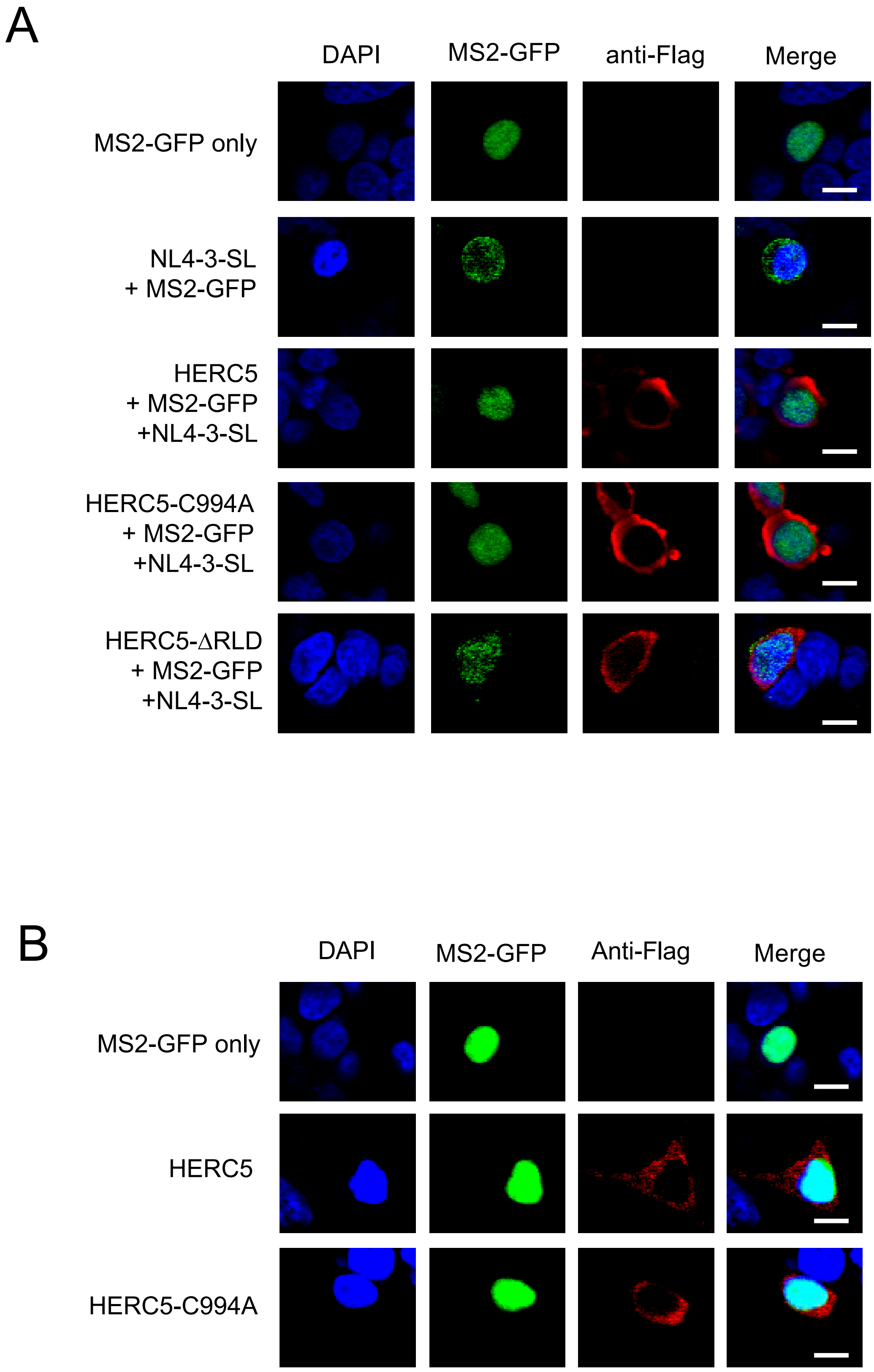

Supplementary Figure 1

Supplemental Data

Figure S2: Alignment of protein sequences for HERC5 mammalian species.

HUMAN	1	MERRSRRKSRRNGRSTAGKAAAT	QPAKSPGAQL	WLFPSAAGL	HRALLRRV	EVTRQLCCSP	60
CHIMPANZEE	1	MERRSRRKSRRNGRSTAG-----	KAAASQPAK-	---------	--------	--------SP	29
GORILLA	1	MERRSRRKSRRNGRSTAGKAAAS	QPAKSPGAQL	WLFPSAAGL	HRALLRRA	EVTRQLCCSP	60
MARMOSET	1	MERRSGRKSRRDRRSVAGQAAAS	QPAKSPGAQL	WLFPSTAGL	RRALLRSV	EATRQICCSS	60
BABOON	1	MERRSRRKSRRGGRSTAGQTAAS	QPAKSPDAQL	WLFPSAAGF	YRALLRRA	EVTRQICCSP	60
SQUIRREL MONKEY	1	MERRSRRKSRHDRRSVAGRAAAS	QPAKSPGAQL	WLFPSTAGL	RRTLLRRV	EATRQICCSS	60
GIBBON	1	MERRSRRKSRRNGRSTAGAAAAS	QPAKSPGAQL	WLFPSAAGF	HRALLRRA	EVTRQICCSP	60
HORSE	1	-----------------------	----------	---------	--------	-----MYCTR	5
PANDA	1	[4]PRRRARG----GVRPVSGPVP--[3]	PPXXXXXXXX[1]	WLFPSAAGL	RRALSQRM	EATRQMCCTR	62
SHEEP	1	[4]PRRRSRG----GARPVSGRVPES[1]	PSASRPSASR[14]	WLFPSAAGL	RSALSKRP	EATRQMCCTR	75
COW	1	[4]PRRRSRG----GARPVPGRVSES[1]	PPASRPSA--[11]	WLFPSAAGL	RSALSKRP	EATRQMCCTR	70
DOG	1	[236]PPRRARG----GARPAPGPRPAP[1]	RPAEPPGTQL	WLFPSAAGL	RRALPPRA	EVAGADGARR	293
CAT	1	[32]PKPLSTGVQVAKARPGTPPQPCP[15]	PPKQHVASPA[1]	ELGRSRGGY[65]	RRFEPQRX[6]	EATRQMCCTR	179

HUMAN	61	GRL---AVLE--RGGAGVQVHQLL	AGSGGA-RTPKCIKLGKNMKIHSVDQGAE-HMLILSSDGKPFEYDnYSMKHLR	130
CHIMPANZEE	30	GTQ---LWLF--PSAAGLQ--RAL	LRRVEV-TRQLCCSPGRLAVLERGGAGVQvHQLLAGSGG--------------	84
GORILLA	61	GRL---AVLE--RGGAGVQVHQLL	AGSGGA-RTPKCIKLGKNMKIHSVDQGAE-HMLILSSDGKPFEYDnYSMKHLR	130
MARMOSET	61	GRL---AFLE--RGGAGVQVHQLL	PGSDGL-RTPKCIKLGKNMKIHSMDLGAD-HMLILSSDGKPFEYDnYSMKHLR	130
BABOON	61	RRL---AVLE--RGGAGVQVHQVL	AGSGGA-RTPKCIKLGKNMKIHSVDQGAD-HMLILSSDGKPFEYD-YSMKHLR	129
SQUIRREL MONKEY	61	GRL---AFLE--RGGAGVQVHQLL	PGSDGL-RTPKCIKLGKNMKIHSMDLGAD-HMLILSSGGKPFEYDnYSMKHLR	130
GIBBON	61	GRL---AVLE--RGGAVVQVHQLL	PGSGGA-RTPKCIKLGKNMKIHSVDQGAE-HMLILSSDGKPFEYDnYSMKHLR	130

☑	HORSE	6	GRL---AVLE--RAGAGXPVLXL-[1]AGSDGT-RKPKCIKLGRKMKIHSMDQGAE-HLLVLSSDGKPFEYN-YSIEHAR	74
☑	PANDA	63	GRL---VVLE--RGGAGVEVHQLX[9]AGGDPTlQRPACVKLGKKMKIHSVDQGAE-HMLILSSDGKPFEYN-YSIEHAR	141
☑	SHEEP	76	RRL---AVLE--RGGAGVEVHQL-[1]AGSDGA-RKPKCIKLGKKMKIHSMDQGEE-HMLVLSSDGKPFEYK-YSIEHAR	144
☑	COW	71	RRL---AVLE--RGGAGVEVHQL-[1]AGSDGA-RKPKCIKLGKKMKIHSMDQGEE-HMLVLSSDGKPFEYK-YSIEHAR	139
☑	DOG	294	SRQpwpVGLEglEGLEGLEPWRLR[3]--SYSFcDSRECIKLGKKMKIHSVDQGAE-HMLILSSDGKPFEYN-YSIEHAR	369
☑	CAT	180	GRL---VVLE--RGGAGVEVHQL-[1]AGSDGT-RKPKCIKLGKKAKIHSVDQGAE-YMLILSSDGKPFEYN-YSIEHAR	248

☑	HUMAN	131	FESILQEKKIIQITCGDYHSLALSKGGELFAWGQNLHGQLGVGRKFPSTTTPQIVEHLAGVPLAQISAGEAHSMALSMSG	210
☑	CHIMPANZEE	85	---------------------ARTPSGELFAWGQNLHGQLGVGRKFPSTTTPQIVEHLAGVPLAQISAGEAHSMALSMSG	143
☑	GORILLA	131	FENVLQEKKIIQITCGDYHSLALSKGGELFAWGQNLHGQLGVGRKFPSTTTPQIVEHLAGVPLAQISAGEAHSMALSMSG	210
☑	MARMOSET	131	FESILQEKKIIQITCGDYHSLALSKGGELFAWGQNLHGQLGVGRKFPSTTAPQIVEHLAGIPLAQISAGEAHSMALSMSG	210
☑	BABOON	130	SESILQEKKIIQITCGDYHSLALSKGGELFAWGQNLHGQLGVGRKFPSTTTPQIVEHLAGVPLAQISAGEAHSMALSMSG	209
☑	SQUIRREL MONKEY	131	FESILQEKKIIQITCGDYHSLALSKGGELFAWGQNLHGQLGVGRKFPSTTTPQIVEHLSGIPLAQISAGEAHSMALSMSG	210
☑	GIBBON	131	FESILQEKKIIQITCGDYHSLALSKGGELFAWGQNLHGQLGVGRKFPSTTTPQIVEHLAGVPLAQISAGEAHSMALSMSG	210
☑	HORSE	75	FQCVLQEKNIIQITCGDYHSLALSKGGELFAWGQNTYGQLGVGRTFASTPTPQIVEHLSGVPLVQISAGEAHSMALSMSR	154
☑	PANDA	142	FQCILQEKSIIQIACGDYHSLALSKGGELFAWGQNLHGQLGVGRLFASIPTPQAVEHLSGVPLVQISAGKAHSMALSMSG	221
☑	SHEEP	145	FQCILQEKNIIQITCGDYHSLALSKGGELFAWGQNLDGQLGVGRIFASTSTPEIVENLSGVPLVQISAGEAHSMALSMSG	224
☑	COW	140	FQCILQEKNIIQITCGDYHSLALSKGGELFAWGQNLDGQLGVGRIFASTSTPEIVENLSGVPLVQISAGEAHSMALSMSG	219
☑	DOG	370	FQCILQEKSIIQITCGDYHSLALSKGGELFAWGQNLHGQLGVGRLFTSIPTPQLVEHLSGVPLVQISAGKAHSMALSMSG	449
☑	CAT	249	FQCILQEKXIIQITCGDYHSLALSKGGELFAWGQNLHGQLGIGSQFASIAIPQIVENLSGIPLVQISAGKAHSMALSMSG	328

☑	HUMAN	211	NIYSWGKNECGQLGLGHTESKDDPSLIEGLDNQKVEFVACGGSHSALLTQDGLLFTFGAGKHGQLGHNSTQNELRPCLVA	290

☑ CHIMPANZEE	144	NIYSWGKNEFGQLGLGHTESKDYPSLIEGLDNQKVEFVACGGSHSALLTQDGLLFTFGAGKHGQLGHNSTQNELRPCLVA	223
☑ GORILLA	211	NIYSWGKNEFGQLGLGHTESKDYPSLIEGLDNQKVEFVACGGSHSALLTQDGLLFTFGAGKHGQLGHNSTQNELRPCLVA	290
☑ MARMOSET	211	NIYSWGKNEFGQLGLDHTESKDSPSLIEALDNQKVEFLACGGSHTALLTQDGLLFTFGAGKHGQLGHNSTQNELRPCLVA	290
☑ BABOON	210	NVYSWGKNEFGQLGLGHTESKDSPSLIEALDNQKVEFLACGGSHSALLTQDGLLFTFGAGKHGQLGHNSTQNELRPCLVA	289
☑ SQUIRREL MONKEY	211	NIYSWGKNEFGQLGLDHTESKDSPSLIEALDNQKVEFLACGGSHTALLTQDGLLFTFGAGKHGQLGHNSTQNELRPCLVA	290
☑ GIBBON	211	NIYSWGKNEFGQLGLGHTESKDYPSLIEALDNQEVEFLACGGSHSALLTQDGLLFTFGAGKHGQLGHNSTQNELRPCLVA	290
☑ HORSE	155	NVYSWGRNDCGQLGLGHTNNKDSPSLIEALDNQEVEFLACGGSHTALLTKDGLVFTFGAGKCGQLGHNSTQNELTPCLVT	234
☑ PANDA	222	NVYSWGRNDLGQLGLGHTDGEDFPSLIETLDNQQVEFLACGGSHTALLTTDGLVFTFGAGKYGQLGHNSTQNELRPRLVT	301
☑ SHEEP	225	NVYSWGRNDCGQLGLGHTYNKDSPSCIEALDDQKVEFLACGGSHTALLTKSGLVFTFGDGKYGQLGHNSTQNELRPCLVA	304
☑ COW	220	NVYSWGRNDCGQLGLGHTYNKESPSCIEALDDQKVEFLACGGCHTALLTKSGLVFTFGDGKYGQLGHNSTQNELRPRLVT	299
☑ DOG	450	NIYSWGRNDLGQLGLGHTNGEDFPSLIEALDNQKVEFLACGGSHTALLTKDGLVFTFGAGKYGQLGHNSTQNELRPRLVT	529
☑ CAT	329	NIYSWGRNDFGQLGLGHTDGKDFPSLIEALDNQKVEFLACGGSHTALLTKDGLVFTFGAGKHGQLGHNSTQNELRPRLVT	408

☑ HUMAN	291	ELVGYRVTQIACGRWHTLAYVSDLGKVFSFGSGKDGQLGNGGTRDQLMPLPVKVSSSEELKLESHTSEKELIMIAGGNQS	370
☑ CHIMPANZEE	224	ELVEYRVTQIACGRWHTLAYVSDLGKVFSFGSGKDGQLGNGGTRDQLMPLPVKVSSSEELKLESHTSEKELIMIAGGNQS	303
☑ GORILLA	291	ELVGYRVTQIACGRWHTLAYVSDLGKVFSFGSGKDGQLGNGGTRDQLMPLPVKVSSSEELKLESHTSEKELIMIAGGNQS	370
☑ MARMOSET	291	ELAGNRVTQIACGRWHTLAYVSDLGKVFSFGSGKEGQLGNGGTRDQLIPLPMKVSSNEDLKFESHTSEKELIMIAGENQS	370
☑ BABOON	290	ELVGNRVTQIACGRWHTLAYVSDLGKVFSFGSGKDGQLGNGGTLDQLIPLPVKVSSSEELKLESHTSEKELIMIAGGNQS	369
☑ SQUIRREL MONKEY	291	ELAGNRVTQIACGRWHTLAYVSDLGKVFSFGSGKEGQLGNGGTRDQLIPLPMKVSSNEDLKFESHTSEKELIMIAGENQS	370
☑ GIBBON	291	ELVGNRVTQIACGRWHTLAYVSDLGKVFSFGSGKDGQLGNGGTRDQLIPLPVKVSSSEELKLETHTSEKELIMIAGGNQS	370
☑ HORSE	235	ELVGKRVTQIACGRRHTLAYVSDLGKVFSFGSGEGGQLGNGATHHQLIPLPMKLPSNEELKFESHTSEKELIMIAGGNQS	314
☑ PANDA	302	ELAGNRVTQIACGRRHTLAYVSDLGKVFSFGSGKEGQLGNGGTHNQLIPLPMKLPSNEELKFESRTSDKELVMIAGGNQS	381
☑ SHEEP	305	GLVGNRVTQIACGRQHTLAYVSDMGKVFSFGSGKEGQLGNGGTCNQLMPRPMKLPSSEELKSESSTSVKELIMVAGGNQS	384

COW	300	GLVGNRVTQIACGRQHTLAYVSDTGKVFSFGSGKEGQLGNGGTRNQLIPRPMKLLSSEELKSESSTSVKELIMVAGGNQS	379
DOG	530	ELAGNRVTQVACGRWHTLAYVSDLGKVFSFGSGKEGQLGNGGTHNQLIPLPMKLPSNEELKFESRTSEKELIMIAGGNQS	609
CAT	409	ELAGNRVTQIACGRQHTLAYVSDLGKVFSFGSGKEGQLGNGGTHNQLIPLPMKLPSNEELTFESRASEKELIMIAGGNQS	488

HUMAN	371	ILLWIKKENSYVNLKRTIPTLNEGTVKRWIADVETKRWQSTKREIQEIFSSPACLTGSFLRKRRTTEMMPVYLDLNKARN	450
CHIMPANZEE	304	ILLWIKKENSYVNLKRTIPTLNEGTVKRWIADVETKRWQSTKREIQEIFSSPACLTGSFLRKRRTTEMMPVYLDLNKARN	383
GORILLA	371	ILLWIKKENSYVNLKRTIPTLNEGTVKRWIADVETKRWQSTKREIQEIFSSPACLTGGFLRKRKDEK----FSSLNFGRT	446
MARMOSET	371	ILLWIKKENSYVNLRRTILTLNEGTIKRWIADVETKRWQSTKREIQEIFSSPACLSGSFLRKRRTTEMLPVYLDLSKARN	450
BABOON	370	ILLWIKKENSYVNLKRTIPTLNEGTVKRWIADVETKRWHSTKREIQEIFSSPACLTGSFLRKRRTAEMMPVYLDLNKARN	449
SQUIRREL MONKEY	371	ILLWIKKENSYVNLRRTILTLNEGTVKRWIADVETKRWQSTKREIQEIFSSPACLSGSFLRKRRTTEMMPVYLDLSKART	450
GIBBON	371	ILLWIKKENSYVNLKRTIPTLNEGTVRRWIADVETKRWQSTKREIQEIFSSPACLTGSFLRKRRTTEMMPVYLDLNKARN	450
HORSE	315	ILLWMEKENPYVNLRRKIPTLNEGTVKRWIADVGTKKWQNTKREIKEIFSSPACLTGSFLRERITAETMSVHLNINKARD	394
PANDA	382	VLLWMEKKNPYVNLRRKIPTLNEGTVKRWIADVGTKQWQNTKREIREIFSSSACLTGSFLREREAAETMLIHLDLNKARN	461
SHEEP	385	ILMWMEKENSYVNLRRKIPTLNEGTVKRWIADVGTKQWQNTKREIREIFSSPACLTGSFLRERIDGEMISIHLDLNAARN	464
COW	380	ILIWMEKENSYVNLRRKIPTLNEGTVKRWIADVGTKRWQNTKREIREIFSSPACLTGSFLRERIDGKMISIHLDLNTARN	459
DOG	610	ILLWMGKKNPYVNLRRRIPTLNEGTVKRWIADVGTKQWQNTKREIREIFSSPACLIGSFLKERIAAETMLIHLDLNKARN	689
CAT	489	ILLWMGKKNSYVNLRRKIPTLNEGTVKRWIADVGTKQWQNTKKEIREIFSSPACLTGSFSRERRAAETMPVHLDLNKARN	568

HUMAN	451	IFKELTQKDWITNMITTCLKDNLLKRLPFHSPPQ	EALEIFFLLPECPMMHIS	NNWESLV-VPFAKVVCKMSDQ	522
CHIMPANZEE	384	IFKELTQKDWITNMITTCLKDNLLKRLPFHSPPQ	EALEIFFLLPECPVMHIS	NNWESLV-VPFAKVVCKMSDQ	455
GORILLA	447	TAPLGVRGE----MITTCLKDNLLKRLPFHSPPQ	EALEIFFLLPECPVMHIS	NNWESLV-VPFAKVVCKMSDQ	514
MARMOSET	451	IFKELIQKDWITNTITTCLKDNLLKRLPFHSPYQ	EALEVFFLLPECPVMHNS	NNWESIV-VAFAKAVCKMSDQ	522
BABOON	450	IFKELTQKDWITNMVTTCLKDNLLKRLPFHSPHQ	EALEVFFLLPECPVMHLS	NNWESLV-VPFAKVVCKMSDP	521

SQUIRREL MONKEY	451	IFKELTQKDWITNTITTCLKDNLLKRLPFHSPYQ EALEVFFLLPECPVMHNS NNWESIV-VAFAKAVCKMSDQ	522
GIBBON	451	IFKELTQKDWITNMITTCLKDNLLKRLPFRSPPQ EALEVFFLLPECPVMHIS NNWESLV-VPFAKVVCKMSDQ	522
HORSE	395	TFKELTQKDWITNMITTCLRDNLIKNLPFHSPHR EALEVFFLLPECPVMHDY NNWESLV-VPFAEAICKMSNQ	466
PANDA	462	AFKELTQKDWIANTITTCLKDNLLKNLPFHSPHQ[21]ESLVVPFAEVVCAMSDQS[5]EYWASLQdAAFIRLVQMFKRA	560
SHEEP	465	TFKELTQKDWITNTITSCLRDNLLKNLPFNSPHQ EALEVFFLLPECPVMHDS NNWESLV-VPFAEAVNKMNNQ	536
COW	460	TFKELTQKDWITNTITSCLKDNLLKNLPFNSPHQ EALEVFFLLPECPVMHDS NNWESLV-VPFAEAVCKMNDQ	531
DOG	690	DFKELTQKDWIANMITTSLKDNLLKNLPFHSPHQ[21]ESLVVPFAEAICAMSDQS[5]EYWASLQeAAFIRLVQMFKRA	788
CAT	569	AFKELTQKDWIANTITTCLKDNLLKNLPFHSPHQ[21]ESLVVPFAEAVCAMSNQS[5]EYWASLQePAFSRLVQMFKGA	667

HUMAN	523	SSLVLEEYWATLQESTFSKLVQMFKTAVICQLD-YWDESAEENGNVQ--ALLEMLKKLHRVNQVKCQLPESIFQVDEL-L	598
CHIMPANZEE	456	SSLVLEEYWATLQESTFSKLVQMFKTAVICQLD-YWDESAEENGNVQ--ALLEMLKKLHRVNQVKCQLPESIFQVDEL-L	531
GORILLA	515	SSLVLEEYWATLQESTFSKLVQMFKTAVICQLD-YWDESAEENGNVQ--ALLEMLKKLHRVNQVKCQLPESIFQVDEL-L	590
MARMOSET	523	SSLVLEEYWATLQESTFSKLVQMFKTAVICQLD-YWNENAEENGNVQ--ALLEMLKKLHRVNQMKCLLPESIFQVDEL-L	598
BABOON	522	SSLVLEEYWATLQESTFSKLVQMFKTAVVCQLD-YWDESAEENGNVQ--ALLEMLKKLHRVNQMKCQLPESIFQVDEL-L	597
SQUIRREL MONKEY	523	SSLVLEEYWATLQESTFSKLVQMFKTAIICQLD-YWNENAEENGNVQ--ALLEMLKKLHRVNQMKCLLPESIFQVDEL-L	598
GIBBON	523	SSLVLEEHWATLQESTFSKLVQMFKTAVICQLD-YWDESAEENGNVQ--ALLEMLKKLHRVNQMKYQLPESIFQVDEL-L	598
HORSE	467	SLGVLEEYWASLQESAFSKLVQMFKRAMGAQLH-YWSESVDSNCHVR--ALLEVLKRLHRVNQSNCQVPENIFEVSEL-T	542
PANDA	561	IT---------------------------AQLH-YWTESSENN--YHvkALLEILKKLHRVNQAVCQLPENIFKINELtH	610
SHEEP	537	MS---------------------------RVLEeYWASLKESP--FI--NLVQMFKRA----------------IVAQlH	569
COW	532	MS---------------------------GVLEeYWASLKESI--FI--NLVQMFKRA----------------VIAQlH	564
DOG	789	VT---------------------------AQLH-YWTESSENN--YHvkALLEILKKLHRVNQAKCQLPENIFKVNELtH	838
CAT	668	IT---------------------------AQLH-YWTESSEDN--CHvkALLEMLRKLYRVNQAKCQLPENIFKINELtQ	717

HUMAN	599	HRLNFFVEVCRRYLWKMTVDAS-ENV---QCCVIFSHFPFIFNNLSKIKLLHTDTLLKIESKKHKAYLRSAAIEEERESE	674
CHIMPANZEE	532	HRLNFFVEVCRRYLWKMTVDTS-ENV---QCCVIFSHFPFIFNNLSKIKLLHTDTLLKIEGKKHKAYLRSAAIEEEGESE	607
GORILLA	591	HRLNFFVEVCRRYLWKMTVDTS-ENV---ECCVIFSHFPFIFNNLSKIKLLHTDTLLKIEGKKHKAYLRSAAIEEERESE	666
MARMOSET	599	YRLDFFVEVCRRYLWKMTVDTL-ENV---GCCVIFSHFPFIFNNLSKIKLLRTDTLVKIQGKKHKAYFRWAAVEEERESE	674
BABOON	598	YRLNFFVEVCRRCLWKMTVDTS-ENA---GCWVIFSHFPFIFNHLSKIKLLHTDTLLKIEGKKHKAYLMSAAIEEERESE	673
SQUIRREL MONKEY	599	YRLDFFVEVCRRYLWKMTVDTL-ENV---GCCVIFSHFPFIFNNLSKIKLLRTDTLVKIQGKKHKAYFRWAAVEEERESE	674
GIBBON	599	HRLNFFVEVCRRYLWKMTVDTS-ENV---ECCVIFSHFPFIFNNLSKIKLLHTDTLLKIEGKKHKAYLRSAAIEEERESE	674
HORSE	543	HWLDFYGDVYKRSAWKMNSDTS-RDS-----PVVFSHFPFIFNILSKIKLLYADSLLKIWEKEQRRFMTLDGNVDPGEPE	616
PANDA	611	W-LDFYGDAYRRSAWK---INS-DASfdtQYPVIFSHFPFIFNILSKIKLLYADSLLKIQEKKFRACMQLAGLVEQGGSE	685
SHEEP	570	Y-------------WT---ESS-ENN---------NHIKALLEVLKKL----------YREKKFQACMRLAGIVDQEGSA	613
COW	565	Y-------------WT---ESS-ENN---------SHIKALLEVLKKL----------YREKKFRACMRLAGIVDQERSA	608
DOG	839	W-LDFYGDAYRRSSWK---VNS-DTSvgtQYPVIFSHFPFIFNILSKIKLLYADSLLKIQEKKFRACMRLAGIMEQGGSQ	913
CAT	718	W-LDFYGDAYRRSSWR---VNNlDTSvdtPYPVIFSHFPFIFNILSKIKLLYADSLLKIQEKKFRACMRLAGVVEHGRSE	793

HUMAN	675	FALRPTFDLTVRRNHLIEDVLNQLSQFENEDLRKELWVSFSGEIGYDLGGVKKEFFYCLFAEMIQPEYGMFMYPEGASCM	754
CHIMPANZEE	608	FALRPTFDLTVRRNHLIEDVLNQLSQFENEDLRKELWVSFSGEIGYDLGGVKKEFFYCLFAEMIQPEYGMFMYPEGASCM	687
GORILLA	667	FALRPTFDLTVRRNHLIEDVLNQLSQFENEDLRKELWVSFSGEIGYDLGGVKKEFFYCLFAEMIQPEYGMFMYPEGGSCM	746
MARMOSET	675	FALLPTFDLTVRRNHLIEDVLNQLSQFENEDLRKELWVSFSGEIGYDLGGVKTEFFYCLFEEMTQPEYGLFMYPEGASCM	754
BABOON	674	FALMPTFDLTVRRNHLIEDVLNQLSQFENEDLRKELWVSFSGEIGYDLVGVKREFFYCLFEEMIQPEYGMFMYPEGASCM	753
SQUIRREL MONKEY	675	FALLPTFDLTVRRNHLIEDVLNQLSQFENEDLRKELWVSFSGEIGYDLGGVKTEFFYCLFEEMTQPEYGLFMYPEGASCM	754
GIBBON	675	FALRPTFDLTVRRNHLIEDVLNQLSQFENEDLRKELWVSFSGEIGYDLGGVKKEFFYCLFEEMIQPEYGMFMYPEGASCM	754
HORSE	617	LALSSSFSLTVRRSHLIEDVLNHLNRFENEDLRRELLVSFSGEIPLDYGGVRAEFFHCLFEELTQPEYGLFTYPEEASYM	696
PANDA	686	LALLPTFNLTVRRTHLIEDVLSHLSQFENEDLRRELMVSFSGEIGHDSGGVKVEFFHCLFEEMTRPEYGMFTYPEEASYM	765

☑	SHEEP	614	LSLLPTFNLIVRRSHLIEDVFNQLNQFENEDLRRELMVSFSGEIGYDFGGVRAEFFYCLFQEMTRPEYGMFTYPEEASYM	693
☑	COW	609	LSSLPTFNLIVRRSHLIEDVFSQLNQFENEDLRRELMVSFSGEIGYNFGGVRAEFFYCLFQEMTRPEYGMFTYPEEASYM	688
☑	DOG	914	LALLPTFNLTVRRSHLIEDVLNHLNQFENEDLRRELMVSFSGEIGHDSGGVKVEFFHCLFEEMTRPEYGMFTYPEDASYM	993
☑	CAT	794	LSLLPTFNLTVRRNHLIEDVLSHLNQFENEDLRRELMVSFSGEIGHDSGGVKVEFFHCLFEEMTRPEYGMFTYPEEASYM	873

☑	HUMAN	755	WFPVKPKFEKKRYFFFGVLCGLSLFNCNVANLPFPLALFKKLLDQMPSLEDLKELSPDLGKNLQTLLDDEGDNFEEVFYI	834
☑	CHIMPANZEE	688	WFPVKPKFEKKRYFFFGVLCGLSLFNCNVANLPFPLALFKKLLDQMPSLEDLKELSPDLGKNLQTLLDDEGDNFEEVFYI	767
☑	GORILLA	747	WFPVKPKFEKKRYFFFGVLCGLSLFNCNVANLPFPLALFKKLLDQMPSLEDLKELSPVLGKNLQTLLDDEGDNFEEVFYI	826
☑	MARMOSET	755	WFPVKPKFEKKRYFFFGVLCGLSLFNCSVANLPFPLALFKKLLDQMPSLEDLKELSPDLGKNLQTILDDEGDNFEEVFYI	834
☑	BABOON	754	WFPVRPXIEKKRYFFFGLLCGLSLFNCNVANLPFPLALFKKLLDQMPSLEDLKELSPDLGKNLQTLLDDEGDNFEEVFYI	833
☑	SQUIRREL MONKEY	755	WFPVKPKFEKKRYFFFGVLCGLSLFNCSVANLPFPLALFKKLLDQMPSLEDLKELSPDLGKNLQTILDDEGDNFEEVFYI	834
☑	GIBBON	755	WFPVKPKFEKKRYFFFGVLCGLSLFNCNVANLPFPLALFKKLLDQMPSLEDLKELSPDLGKNLQTLLDDEGDNFEEVFYI	834
☑	HORSE	697	WFPVRPKFEKKSYFFFGVLCGLCLFNCNVANIPFPLALFKKLLDQTPSLEDLKELSPVFGRSLQTLLDDEGEDFGEVFYI	776
☑	PANDA	766	WFPVRPKFEKKRYFFFGVLCGLSLFNFNVANIPFPLALFKKLLNQTPSLEDLKELSPVLGKSLQTLLDDEGDDFGEVFYI	845
☑	SHEEP	694	WFPVRPKFEKKSYFFFGLLCGLSLFNCNVADIPFPLALFKKLLDQTPSLEDLKELSPVLGESLQTLLDDDGDDLEEVFHI	773
☑	COW	689	WFPVRPKFEKKSYFFFGLLCGLSLFNCNVADIPFPLALFKKLLDQTPSLEDLKELSPVLGESLQTLLDDDGDDLEEVFHI	768
☑	DOG	994	WFPVTPKFEKKRYFFFGVLCGLSLFNFNVANIPFPLALFKKLLDQTPSLEDLKELSPVLGKSLQTLLDDEGDDFGEVFLI	1073
☑	CAT	874	WFPVRPKFEKKRYFFFGVLCGLSLFNFNVANIPFPLALFKKLLNQAPSLEDLKELSPVLGKSLQTLLDDEGDDFGEVFYI	953

☑	HUMAN	835	HFNVHWDRNDTNLIPNGSSITVNQTNKRDYVSKYINYIFNDSVKAVYEEFRRGFYKMCDEDIIKLFHPEELKDVIVGNTD	914
☑	CHIMPANZEE	768	HFNVHWDRNDTNLIPNGSSITVNQTNKRDYVSKYINYIFNDSVKAVYEEFRRGFYKMCDEDIIKLFHPEELKDVIVGNTD	847
☑	GORILLA	827	HFNVHWDRNDTNLIPNGSSITVNQTNKRDYVSKYINYIFNDSVKAVYEEFRRGFYKMCDEDIIKLFHPEELKDVIVGNTD	906
☑	MARMOSET	835	YFNVHWDRNDTDLIPNGRNIIVNQTNKRDYVSKYIDYIFNDSVKVVYEEFRRGFYKMCEEDIIKLFHPEELKDVIVGNTY	914

BABOON	834	HFNVHWDRNDTNLIPNGSSIIVNQTNKRDYVSKYIDYIFNDSVKAVYEEFRRGFYKMCDEDIIKLFHPEELKDVIVGHTD	913
SQUIRREL MONKEY	835	HFNVHWDRNDTDLIPNGSNIIVNQTNKRDYVSKYIDYIFNDSVKVVYEEFRRGFYKMCEEDIIKLFHPEELKDVIVGNTD	914
GIBBON	835	HFNVHWDRNDTNLIPNGSSIIVNQTNKRDYVSKYIDYIFNDSVKAVYEEFRRGFYKMCDEDIIKLFHPEELKDVIVGNTD	914
HORSE	777	HFTVHWDRNAEELIPNGSDIIVDQTNKRDYVSKCVNYIFNISIKAVYEEFQRGFYKVCDKEIIGFFHPAELKDVIIGNTD	856
PANDA	846	YFNVHWDKNDIDLIPNGSCVIVDQTNKRDYVSKCVSYIFNISVKALYEEFQRGFYKVCDKDIIEFFHPEELKDVIIGNTD	925
SHEEP	774	HFNVHWDKNDVDLIPDGSHIIVDQTNKRDYVSKYVNYIFNISVKAVYEEFQRGFYKVCDKEIIEFFHPEQLKDVIIGNTD	853
COW	769	HFNVHWDKNDVDLIPDGSHVIVDQTNKRDYVSKYVNYIFNISVKAVYEEFQRGFYKVCDKEIIEFFHPEQLKDVIIGNTD	848
DOG	1074	YFNVHWDKNDVDLIPNGSGIIVDQTNKRDYVSKYVNYIFNISVKAVYEEFQRGFYKVCDKDIIEFFHPEELKDVVIGNTD	1153
CAT	954	YFNVHWDKNDVDLIPNGRCIIVDQTNKRDYVSKCVNYIFNISVKAVYEEFQRGFYKVCEKDIIEFFHPEELKDVIVGNTD	1033

HUMAN	915	YDWKTFEKNARYEPGYNSSHPTIVMFWKAFHKLTLEEKKKFLVFLTGTDRLQMKDLNNMKITFCCPESWNERDPIRALTC	994
CHIMPANZEE	848	YDWKTFEKNAHYEPGYNSSHPTIVMFWKAFHKLTLEEKKKFLVFLTGTDRLEMKDLNNMKITFCCPESWNERDPIRALTC	927
GORILLA	907	YDWKTFEKNACYEPGYNSSHPTIVMFWKAFHKLTLEEKKKFLVFLTGTDRLQMKDLNNMKITFCCPESWNERDPIRALTC	986
MARMOSET	915	YDWKTFEKNAHYKPEYDSSHPTIVMFWEAFHKLTLEEKKKFLVFLTGTDRIQIKDLKNMKITFCCPENWNEREPMRALTC	994
BABOON	914	YDWKTFEKNARYEPGYNSSHPTIVMFWKAFHKLTLEEKKKFLVFLTGTDRLQTKDLKNMKITFCCPESWNERDPMRALTC	993
SQUIRREL MONKEY	915	YDWKTFEKNAHYKPEYDSSHPTIVMFWEAFHKLTLEEKKKFLVFLTGTDRIQMKDLKNMKITFCCPENWNERDPMRALTC	994
GIBBON	915	YDWKTFEKNARYEPGYNSSHPTIVMFWKAFHKLTLEEKKQFLVFLTGTDRLQMKDLNNMKITFCCPESWNERDPMRALTC	994
HORSE	857	YDWETFEKNARYAGGYDSSHPTIVMFWEALHKLTLEEKKKFLVFLTGTDRLQVKGVKNMKITFCCPEHLDEKDPIRAQTC	936
PANDA	926	YDWETFEKNAHYEEGYDNSHPTIVMFWKALHKLTLEEKKKFLVFLTGTDRIQVKGLKNMKITFCCPENVNEKDPIRAQTC	1005
SHEEP	854	YDWETFEKNAHYEHGYDSSHPTIVMFWKALHKLTLEEKKKFLVFLTGTDRIQVKGVKNMKITFCCPETLNEKDPIRAQTC	933
COW	849	YDWETFEKNAHYEHGYDSSHPTIVMFWKALHKLTLEEKKKFLVFLTGTDRIQVKGLKNMKITFHCPENLNEKDPIRAQTC	928
DOG	1154	YDWETFEKNAHYEEGYDNSHPTIVMFWKALHKLTLEEKKKFLVFLTGTDRIQVKGLKNMKITFCCPENVNEKDPIRAQTC	1233
CAT	1034	YDWETFEKNAHYEEGYDNSHPTIVMFWKALHKLTLEEKKKFLVFLTGTDRIQVKGLKNMKITFCCPENMNEKDPIRAQTC	1113

☑	HUMAN	995	FSVLFLPKYSTMETVEEALQEAINNNRGFG	1024
☑	CHIMPANZEE	928	FSILFLPKYSTMETVEEALQEAINNSRGFG	957
☑	GORILLA	987	FSVLFLPKYSTMETVEEALQEAINNNRGFG	1016
☑	MARMOSET	995	FSVLFLPKYSTMERVEEALQVAINNNRGFG	1024
☑	BABOON	994	FSVLFLPKYSTMETVEEALQVAINNNRGFG	1023
☑	SQUIRREL MONKEY	995	FSVLFLPKYSTMERVEEALEVAINNNRGFG	1024
☑	GIBBON	995	FNVLFLPKYSTMETVEEALQEAINNSRGFG	1024
☑	HORSE	937	FSVLYLPKYSTMERVEEALQVAINNNRGFG	966
☑	PANDA	1006	ISVLYLPKYSTMERVEEALQVAINNSRGFG	1035
☑	SHEEP	934	FSVLYLPKYSTMERVEEALQVAINNSRGFG	963
☑	COW	929	FSVLYLPKYSTMERVEEALQAAINNKRGFG	958
☑	DOG	1234	ISVLYLPKYSTMERVEEALQVAINNSRGFG	1263
☑	CAT	1114	ISVLYLPKYSTMERVEEALQVAINNSRGFG	1143

1	11	21	31	41
MERRSRRKSR	RNGRSTAGKA	AATQPAKSPG	AQLWLFPSAA	GLHRALLRRV
51	61	71	81	91
EVTRQLCCSP	GRLAVLERGG	AGVQVHQLLA	GSGGARTPKC	IKLGKNMKIH
101	111	121	131	141
SVDQGAEHML	ILSSDGKPFE	YDNYSMKHLR	FESILQEKKI	IQITCGDYHS
151	161	171	181	191
LALSKGGELF	AWGQNLHGQL	GVGRKFPSTT	TPQIVEHLAG	VPLAQISAGE
201	211	221	231	241
AHSMALSMSG	NIYSWGKNEC	GQLGLGHTES	KDDPSLIEGL	DNQKVEFVAC
251	261	271	281	291
GGSHSALLTQ	DGLLFTFGAG	KHGQLGHNST	QNELRPCLVA	ELVGYRVTQI
301	311	321	331	341
ACGRWHTLAY	VSDLGKVFSF	GSGKDGQLGN	GGTRDQLMPL	PVKVSSSEEL
351	361	371	381	391
KLESHTSEKE	LIMIAGGNQS	ILLWIKKENS	YVNLKRTIPT	LNEGTVKRWI
401	411	421	431	441
ADVETKRWQS	TKREIQEIFS	SPACLTGSFL	RKRRTTEMMP	VYLDLNKARN
451	461	471	481	491
IFKELTQKDW	ITNMITTCLK	DNLLKRLPFH	SPPQEALEIF	FLLPECPMMH
501	511	521	531	541
ISNNWESLVV	PFAKVVCKMS	DQSSLVLEEY	WATLQESTFS	KLVQMFKTAV
551	561	571	581	591
ICQLDYWDES	AEENGNVQAL	LEMLKKLHRV	NQVKCQLPES	IFQVDELLHR
601	611	621	631	641
LNFFVEVCRR	YLWKMTVDAS	ENVQCCVIFS	HFPFIFNNLS	KIKLLHTDTL
651	661	671	681	691
LKIESKKHKA	YLRSAAIEEE	RESEFALRPT	FDLTVRRNHL	IEDVLNQLSQ
701	711	721	731	741
FENEDLRKEL	WVSFSGEIGY	DLGGVKKEFF	YCLFAEMIQP	EYGMFMYPEG
751	761	771	781	791
ASCMWFPVKP	KFEKKRYFFF	GVLCGLSLFN	CNVANLPFPL	ALFKKLLDQM
801	811	821	831	841
PSLEDLKELS	PDLGKNLQTL	LDDEGDNFEE	VFYIHFNVHW	DRNDTNLIPN
851	861	871	881	891
GSSITVNQTN	KRDYVSKYIN	YIFNDSVKAV	YEEFRRGFYK	MCDEDIIKLF
901	911	921	931	941
HPEELKDVIV	GNTDYDWKTF	EKNARYEPGY	NSSHPTIVMF	WKAFHKLTLE
951	961	971	981	991
EKKKFLVFLT	GTDRLQMKDL	NNMKITFCCP	ESWNERDPIR	ALTCFSVLFL
1001	1011	1021		
PKYSTMETVE	EALQEAINNN	RGFG		

Legend:

The selection scale:

1	2	3	4	5	6	7
Positive selection				Purifying selection		

Box 1

Sequences queried

NP_057407.2 = Human
XP_003310459.1 = Chimpanzee
XP_004039179.1 = Gorilla
XP_002745648.1 = Marmoset
XP_003898997.1 = Baboon
XP_003924055.1 = Squirrel monkey
XP_003265940.1 = Gibbon
XP_001915115.2 = Horse
XP_002913645.1 = Panda
XP_004009762.1 = Sheep
NP_001095465.1 = Cow
XP_535652.3 = Dog
XP_003985249.1 = Cat

Supplementary Figure 3
